# Unplugging Asymmetric Synthesis with a Wireless, Self-Pumping Electrochemical Reactor

**DOI:** 10.1021/jacs.5c16187

**Published:** 2025-12-10

**Authors:** Sara Grecchi, Gerardo Salinas, Malinee Niamlaem, Alexander Kuhn, Serena Arnaboldi

**Affiliations:** † Dipartimento di Chimica, 9304 Università degli Studi di Milano, Via Golgi 19, 20133 Milan, Italy; ‡ CNRS, Bordeaux INP, ISM, Univ. Bordeaux, UMR 5255, 33607 Pessac, France

## Abstract

Herein, we report the first miniaturized, wireless electrochemical flow reactor capable of performing both reactant pumping and asymmetric synthesis within a single, integrated device. The reactor consists of a hollow, conductive polymer tube where the outer polypyrrole (Ppy) shell acts as an electromechanical pump, and the inner layer, constituted of a chiral thiophene-based oligomer, serves as the enantioselective catalyst. This integrated design overcomes mass-transport limitations and eliminates the need for external pumps. By employing an alternating current (AC) protocol, we achieve near-quantitative yield (99%) and exceptional enantioselectivity (>99% ee) for the reduction of acetophenone. The system’s utility is showcased across three mechanistically distinct transformations, ketone reduction, sulfide oxidation, and reductive amination, culminating in the direct asymmetric synthesis of Ugi’s amine, the chiral probe used in our mechanistic studies, with high stereocontrol (>99.5% ee). This work introduces a new paradigm for reagent-free, pump-free asymmetric synthesis and provides a validated, predictive model for the rational design of smart, automated chemical manufacturing platforms.

## Graphical abstract

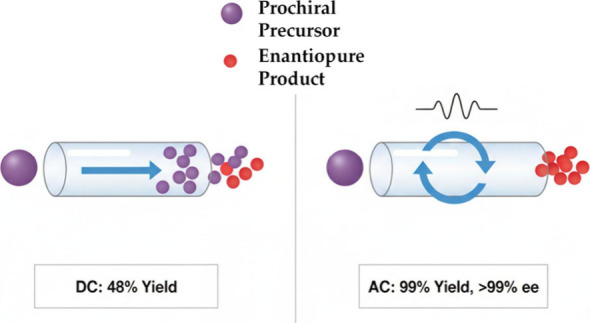

## 1 Introduction

Highly efficient enantioselective synthesis represents one of the most challenging and important goals in modern organic chemistry, as the enantiopure form of a chiral compound is essential for the pharmaceutical, agrochemical, and food industries.^1–9^ Traditional approaches often face significant hurdles. Homogeneous catalysts, while effective, frequently rely on expensive and toxic transition or rare-earth metals and present considerable challenges in product purification.^10–13^ Heterogeneous catalysts offer easier separation and recycling, but their performance is often hampered by slow kinetics and mass transport limitations at the solid–liquid interface.^14,15^

In recent years, organic electrochemistry has experienced a renaissance, championed as a green and sustainable methodology that uses electrons as traceless reagents, operates under mild conditions, and offers tunable control over reactivity and selectivity.^16–19^ The fusion of electrochemistry with continuous flow technology further enhances these benefits, improving mass and heat transfer, increasing safety, and enabling scalability and automation. However, the application of this powerful combination to asymmetric synthesis remains in its nascent stages. Existing strategies typically rely on either chiral mediators in solution, which can be electrochemically fragile, or on chiral-modified electrodes, which often require complex, multicomponent reactor setups with external pumps and sophisticated fluidic control.^20–23^

Here, we overcome these limitations by introducing a single, integrated device that functions as an all-in-one “pump-and-react” system. We leverage the principles of bipolar electrochemistry (BE), a wireless technique where an external electric field induces simultaneous oxidation and reduction at opposite poles of a conducting object, known as a bipolar electrode (BPE).^24–28^ Our BPE is a hollow tube composed of a hybrid conducting polymer. The outer layer, made of polypyrrole (Ppy, Scheme 1(A)), acts as an electromechanical actuator, generating a fluid flow through the tube without any external pumps.^29,30^ The inner side of the tube is functionalized with an “inherently chiral” oligomer 2,2′-bis­[2-(5,2′-bithienyl)]-3,3′-bithianaphthene, named oligo-BT_2_T_4_ (Scheme 1(A)), which provides an exceptionally effective chiral environment for the reaction.^31,32^

**Scheme 1:**
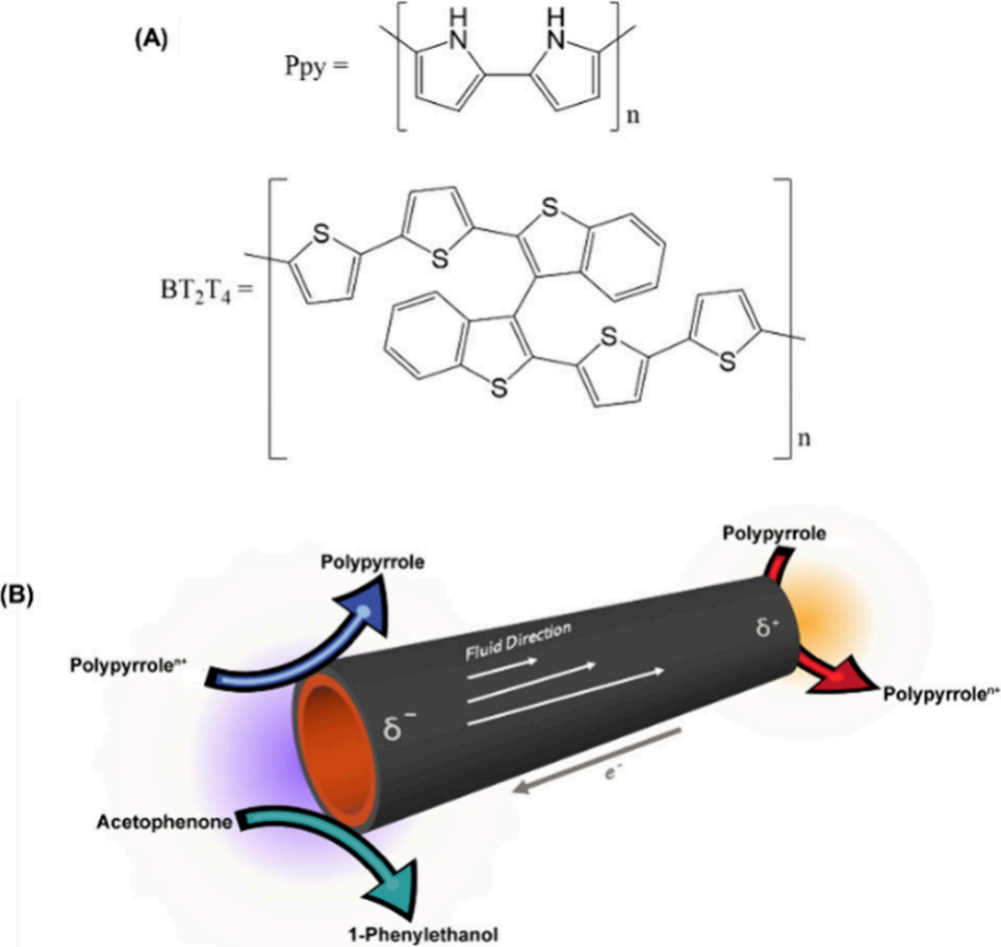
(A) Chemical Structures of Both Ppy and Oligo-BT_2_T_4_ and (B) Schematic Representation of the Enantioselective Electroconversion Process within the Hollow Oligo-BT_2_T_4_/Ppy Hybrid Tube^a^

a The electromechanical pumping mechanism, driven by the wireless oxidation (at the δ^+^ extremity) and reduction (at the δ^–^ side) of doped polypyrrole (Ppy^n^^+^), is depicted, along with the involved redox reactions and the asymmetric polarization. The unidirectional flux drives acetophenone (AP) through the tube, where electrochemical conversion to (*R*)- or (*S*)-phenylethanol (PE) occurs at the cathodic extremity under an applied electric field. The orange and black parts represent the oligo-BT_2_T_4_ and the Ppy film, respectively.

We demonstrate the power of this integrated design for the highly enantioselective reduction of acetophenone (AP) and oxidation of lansoprazole sulfide (Lans-S). Critically, we propose an alternating current (AC) protocol that manipulates the fluid’s residence time within the reactor, driving the reaction to near-quantitative conversion (99% yield) while maintaining close to unit enantioselectivity (>99% ee). This work introduces a new paradigm for reagent-free, pump-free asymmetric synthesis and establishes a foundation for developing the next generation of smart, electrically controlled, and fully automatable systems for fine chemical production.

## 2 Results and Discussion

### 2.1 Design of the Wireless Enantioselective Flux Reactors

The wireless enantioselective flow reactors are based on the synergy between two key components: (i) the electromechanical pumping of a Ppy chassis and (ii) the enantioselective catalytic capabilities of an inner layer of the inherently chiral oligomer, oligo-BT_2_T_4_. The chiral hollow tubes were generated using a previously reported two-step methodology.^29,30^ First, an enantiopure oligomeric film of either (*R*)- or (*S*)-oligo-BT_2_T_4_ was deposited potentiodynamically onto a gold wire template (o̷ = 0.3 mm) from the corresponding monomer. The synthesis of the racemic BT_2_T_4_ monomer via a palladium-mediated Stille reaction and its subsequent resolution into enantiomers by chiral HPLC have been described elsewhere.^31,33^ Following this, a layer of Ppy was galvanostatically grown on top of the chiral oligomer film (*i* = 400 mA, t = 3600 s). The resulting hybrid polymer tube was carefully removed from the wire template and cut to a length of 1 cm (o̷_inner_ ≈ 200 μm, o̷_outer_ ≈ 300 μm and wall thickness = ≈ 100 μm) (Figure S1). When this hollow tube is placed in an electrolyte solution between two feeder electrodes, the applied electric field (ε) induces a polarization potential difference (ΔV) across it, causing it to act as a BPE. If ΔV exceeds the thermodynamic threshold for the relevant redox reactions, the Ppy backbone is oxidized at the anodic pole (δ^+^) and reduced at the cathodic pole (δ^–^) (Scheme 1(B)).^29,30,34^ These redox reactions necessitate a flux of ions to maintain electroneutrality. The Ppy was synthesized with sodium dodecylbenzenesulfonate (DBS) as the dopant anion. Due to its large size, the DBS anion is effectively immobile within the polymer matrix. Consequently, charge compensation is achieved through the movement of smaller, mobile cations (Li^+^ from the LiClO_4_ electrolyte) into and out of the polymer film. This ″cation-driven″ actuation mechanism involves the influx of Li^+^ at the cathodic pole (reduction), causing the polymer to swell, and the efflux of Li^+^ at the anodic pole (oxidation), causing it to shrink. This asymmetric volume change, which leads to a modification of the tube’s inner diameter, has been quantitatively validated as the system’s propulsive mechanism (Table S3). To move beyond analogy to prior devices^34–41^ and provide direct proof in our custom-designed reactor, we have conducted operando experiments. Using Micro-Particle Tracking Velocimetry (μ-PTV), we directly measured the fluid velocity field inside the tube, observing a parabolic velocity profile that is the unequivocal signature of pressure-driven laminar flow. From this data, by applying the Hagen–Poiseuille equation, we calculated the effective pressure gradient generated by the actuator, which increases from 100.5 Pa/m at 1.2 V/cm to 239.4 Pa/m at 1.6 V/cm (Table S4). Furthermore, to establish a direct causal link between Li^+^ ion flux and fluid pumping, synchronized chronoamperometry and time-resolved μ-PTV measurements were performed. As reported in Figure S15 and Table S5, this analysis revealed a direct linear relationship between the total charge passed (quantifying the ion flux) and the volume of fluid displaced. The slope of this line allowed us to define a fundamental metric for our device: a pumping efficiency of approximately 0.16 nL/μC.

This direct experimental evidence, supported by a new electro-chemo-mechanical model, unequivocally confirms that the asymmetric, ion-flux-induced polymer deformation generates the pressure gradient responsible for the observed unidirectional flow. Simultaneously, the inner oligo-BT_2_T_4_ layer acts as the chiral catalyst. A droplet of the prochiral substrate, acetophenone (AP), which is immiscible with the aqueous buffer, is introduced at the cathodic end of the tube. The pumping action draws the AP droplet into the reactor, where it comes into contact with the polarized, chiral inner surface. At the cathodic pole, where the potential is sufficiently negative, the enantioselective reduction of AP to 1-phenylethanol (PE) occurs, guided by the chiral oligomer (Scheme 1(B)). The product is then transported through the tube and expelled at the anodic end. To confirm the feasibility of this integrated process, we evaluated the electrochemical behavior of the components. Cyclic Voltammetry (CV) of a Ppy film showed that the polymer’s redox activity occurs within a potential window of −0.5 to 1 V vs Ag/AgCl (Figure S2). The reduction of AP on Ppy was found to have a threshold potential of approximately −0.3 V vs Ag/AgCl (Figure S2).

Therefore, all necessary redox processes occur within the same potential window. Based on the principles of BE, a minimum polarization potential (Δ*V*_min_) of approximately 1.3 V is required across the 1 cm tube to drive both the Ppy redox cycle and the AP reduction, which corresponds to an external field of 1.3 V/cm.

In addition, AP and the enantiomers of 1-PE were analyzed separately as such through chiral HPLC to evaluate their retention times ().

### 2.2 Wireless Enantioselective Synthesis under Direct Current (DC)

The initial experiments were conducted under the influence of a constant DC electric field. A 1 cm oligo-BT_2_T_4_/Ppy tube was fixed in a bipolar cell containing a pH 4 buffer with 0.2 M LiClO_4_. A 10 μL droplet of neat AP (Figure S4) was placed at the cathodic (δ^–^) end of the tube under an applied field of 1.4 V/cm (Figure 1(A)). The immiscibility of the organic substrate with the aqueous electrolyte facilitates its localized introduction and prevents premature dilution, while also simplifying product collection. After 15 min, the liquid expelled from the anodic (δ^+^) end was collected, extracted with heptane, and analyzed by chiral HPLC. The results unequivocally demonstrate successful asymmetric synthesis. When a tube functionalized with oligo-(*R*)-BT_2_T_4_ was used, the chromatogram showed a new peak corresponding to (*R*)-PE, with a concomitant decrease in the AP peak (Figure 1(B), green line). Conversely, an oligo-(*S*)-BT_2_T_4_ tube selectively produced (*S*)-PE (Figure 1(B), red line). This confirms that the configuration of the product is directly controlled by the chirality of the oligomer film. The enantioselectivity was excellent, with an enantiomeric excess (ee) of approximately 90% achieved for both enantiomers. However, the conversion under these DC conditions was modest, with a product yield of only 48%.

**Figure 1:**
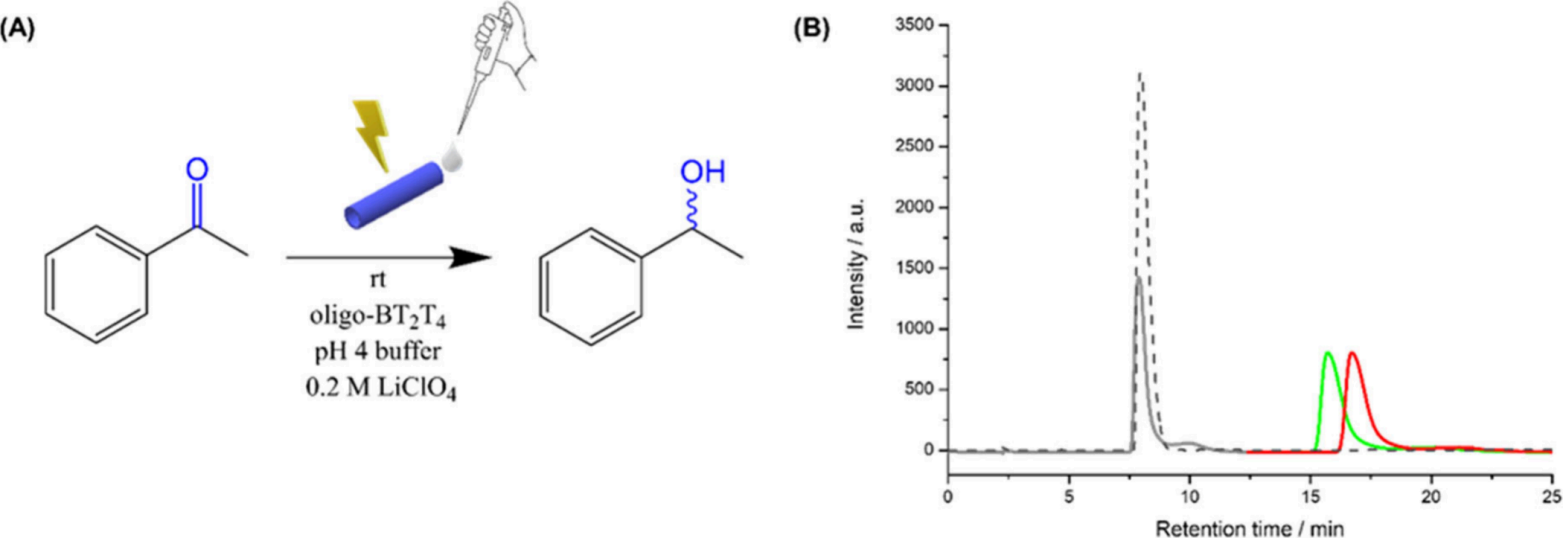
Direct current asymmetric electrosynthesis at a constant electric field of 1.4 V/cm. (A) Schematic representation of the wireless asymmetric electroreduction of AP to PE via the enantioselective flux reactor, along with the corresponding operating conditions. (B) Chromatograms of the fractions collected after 15 min of electrolysis under a constant electric field (1.4 V cm^–1^), using an oligo-(*R*)- or oligo-(*S*)-BT_2_T_4_/Ppy hybrid tube (in green and red, respectively). For comparison, the gray peak corresponds to the unreacted AP following wireless electrosynthesis, while the dark gray dotted line is associated with the chromatogram of the AP before the injection in the hybrid tube.

This can be attributed to the limited residence time of the substrate within the electrochemically active zone. The potential across the BPE varies linearly from its maximum at the poles to zero at the center. Given the AP reduction potential of −0.3 V, only the first 3 mm of the cathodic end of the 1 cm tube possess a sufficiently negative potential to drive the reaction (Figure S5). As the liquid is continuously pumped through the tube in one direction, a significant fraction of the AP passes through this active zone without being converted.

### 2.3 The Mechanism of Enantioselectivity

The exceptional enantioselectivity of the present system, culminating in an ee of over 99%, is a macroscopic outcome rooted in the thermodynamics of the competing reaction pathways. To gain direct, atomic-level insight into the molecular origins of this stereocontrol, a comprehensive Density Functional Theory (DFT) investigation has been carried out to model the stereodetermining transition states.^42^ This computational approach allows visualizing the catalyst-substrate interactions and quantifying the energy differences that govern the reaction’s outcome.^43–45^ The DFT model successfully located the diastereomeric transition states (TS) for the reduction of acetophenone at the chiral oligomer surface. The calculated difference in the Gibbs free energy of activation (ΔΔG^‡^) between the favored and unfavored pathways quantitatively reproduces the experimentally observed enantioselectivity (Table S6). Crucially, the model also provides independent validation of our experimental thermodynamic analysis. The calculated enthalpic (ΔΔH^‡^) and entropic (ΔΔS^‡^) components of the activation barrier are in excellent agreement with the values derived from additional temperature-dependence studies (Eyring analysis, Table S7),^46,47^ confirming that the model accurately captures the fundamental physics of the enantioselection process. The negative ΔΔS^‡^ value, both experimental and calculated, confirms that the favored transition state is significantly more ordered and conformationally restricted.

Beyond quantitative prediction, the computational model provides a clear, chemically intuitive explanation of the observed selectivity. Analysis of the optimized transition state geometries (Figure S16) reveals that stereocontrol arises from a delicate balance of noncovalent interactions.^48^ In the favored transition state leading to the major enantiomer, the acetophenone molecule is oriented to maximize stabilizing C–H···π interactions between its methyl group and a thiophene ring of the oligomer’s helical pocket. In contrast, the transition state leading to the minor enantiomer is destabilized by significant steric repulsion between the substrate’s phenyl group and the catalyst backbone. To demonstrate that this mechanism is related to a general feature of the catalyst and not specific to a single substrate, the same DFT protocol was applied to the enantioselective oxidation of lansoprazole sulfide. The calculations again showed excellent agreement with the experimental ee (>90%), as summarized in Table S6. This successful modeling of two structurally and electronically distinct transformations provides powerful evidence that the chiral recognition mechanism is an intrinsic property of the oligomer’s helical pocket. This detailed computational picture is strongly corroborated by our experimental investigations. The initial host–guest studies using a ferrocene-based probe ((*R*)- or (*S*)-N,N-dimethyl-1-ferrocenylethylamine, (*R*)-Fc or (*S*)-Fc)) quantified by Electrochemical Quartz Crystal Microbalance (EQCM, Figure S7 ^49^) and characterized by solid-state NMR (ssNMR, Figure S6 ^50^) and Magnetic Circular Dichroism (MCD, Figure S8), established the fundamental principles of preferential binding and the formation of electronically distinct, ordered diastereomeric complexes. The DFT model now provides a validated, molecular scale picture of how these principles translate into the actual catalytic transition states. Furthermore, the significant separation in redox potential (Δ*E*_p_) observed in cyclic voltammetry for the enantiomers of the reaction products (Figure S9) provides direct electrochemical evidence for the different energy barriers predicted by the DFT calculations. In summary, the synergistic combination of state-of-the-art DFT calculations and a series of targeted experimental techniques provides a complete and compelling elucidation of the molecular origins of enantioselectivity in this system. The model confirms that the unique, preorganized helical structure of the inherently chiral oligo-BT_2_T_4_ creates a well-defined binding pocket that stabilizes the favored transition state through specific noncovalent interactions, leading to exceptionally high levels of stereocontrol. To provide direct experimental validation of the computational model, and specifically to test the hypothesis that a C–H···π interaction involving the substrate’s methyl group is critical in the stereodetermining step, a kinetic isotope effect (KIE) experiment has been performed.^51,52^ The enantioselective reduction of acetophenone-d_3_ (CD_3_–C­(O)­Ph) was carried out under the established AC protocol and compared directly with its nondeuterated isotopologue. The results were 2-fold and in excellent agreement with the predictions of the model. First, a normal secondary KIE of k_H_/k_D_ = 1.18 was observed, indicating that the reaction proceeds more slowly for the deuterated substrate. Second, and more critically, a significant decrease in stereoselectivity, with the enantiomeric excess dropping from >99% for acetophenone to 97.5% for acetophenone-d_3_ (Table S8) was measured. This dual observation, a slower rate combined with a lower ee, provides powerful, independent evidence that the C–H···π interaction is not merely present, but is a key, rate-influencing factor that preferentially stabilizes the favored transition state. The selective attenuation of this stabilizing interaction upon deuteration reduces the energy gap between the two competing pathways, directly resulting in the observed erosion of enantioselectivity and confirming the intimate involvement of the methyl group in the mechanism of chiral induction.

### 2.4 Alternating Current Asymmetric Synthesis for Enhanced Yield

To overcome the yield limitation of the DC method, we developed an AC protocol (Scheme 2). The core idea is to increase the residence time of the substrate in the active cathodic zone by periodically reversing the polarity of the external electric field. This was achieved by using a programmable function generator connected to the power supply for precise control of the AC waveform and period. As depicted in Scheme S1, when the field is applied in one direction (ε), the liquid flows from the polarized cathode to the polarized anode, with AP reduction occurring at the cathodic end. When the field is inverted (−ε), the roles of the poles are reversed: the former anode becomes the new cathode, and the liquid flows back in the opposite direction. This “forward-and-backward” shuttling of the reactant droplet (Video S1) ensures that the entire volume of the substrate is repeatedly exposed to a polarized, catalytically active surface, maximizing the conversion. We monitored the reaction progress by collecting and analyzing fractions at various time points during a 60 min electrolysis at 1.4 V/cm, with the polarity inverted every 15 min (Figure 2).

**Scheme 2:**
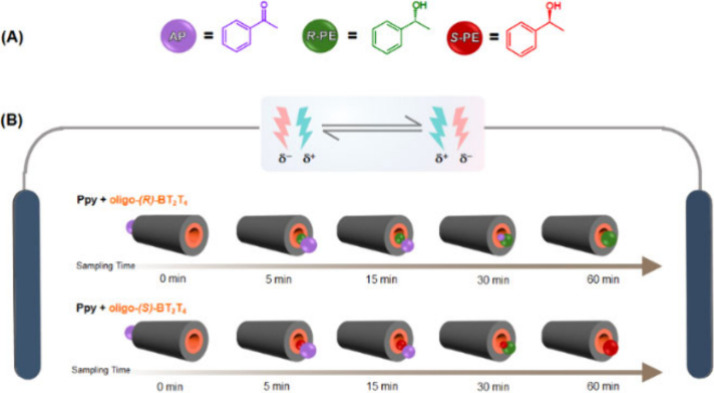
Chemical Structures of the Compounds and AC Asymmetric Synthesis Set-up^a^

a (A) Chemical structures of AP (in purple), (*R*)- and (*S*)-PE (green and red, respectively). (B) Schematic representation of the alternating current method for both oligo-(*R*)- and (*S*)-BT_2_T_4_/Ppy hybrid tubes (top and bottom, respectively), with the corresponding fractions collected at different reaction times.

**Figure 2:**
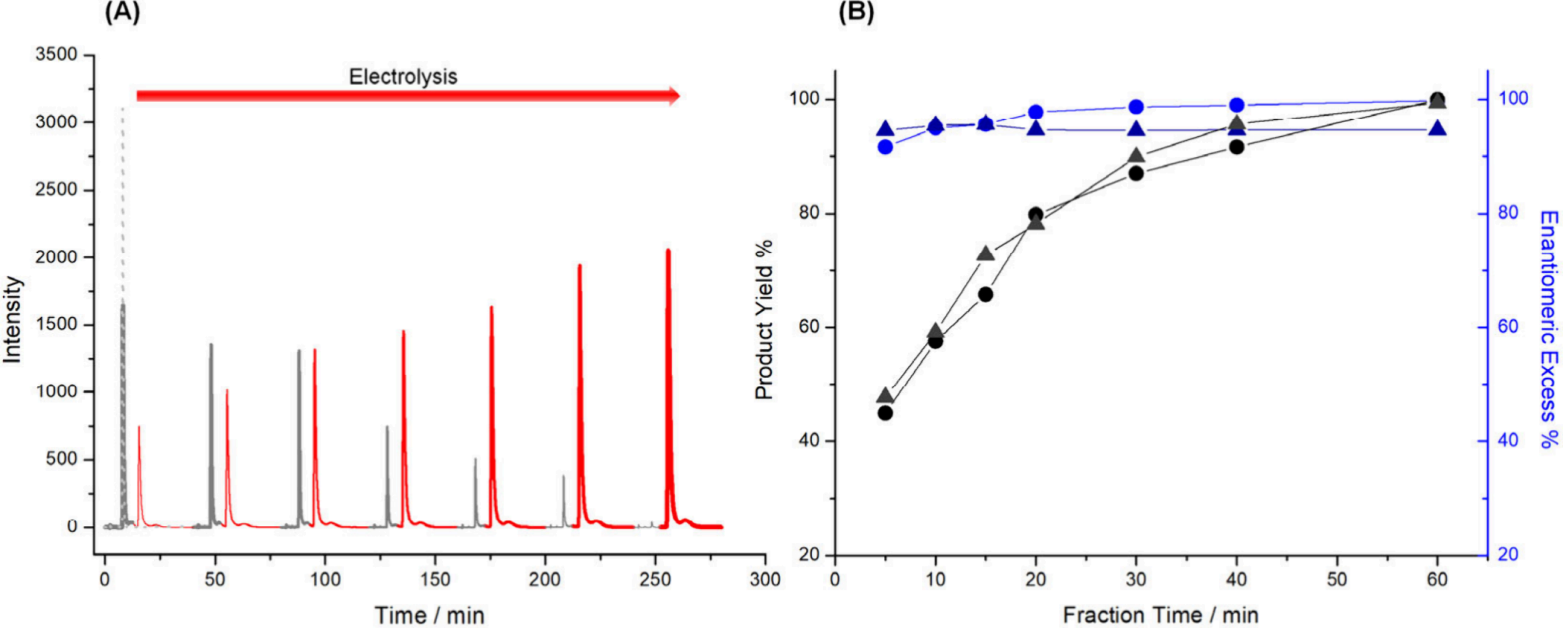
AC asymmetric electrosynthesis at a constant electric field of 1.4 V/cm. (A) Chromatograms of the fractions collected at different reaction times (5, 10, 15, 20, 30, 40, 60 min) from the δ^+^ extremity of an oligo-(*S*)-BT_2_T_4_/Ppy tube. The red color stands for (*S*)-PE, while the gray peaks are related to AP. For comparison, the gray dotted line represents the AP concentration before the injection into the hybrid tube. (B) Enantiomeric excess (in blue) and product yield (in black) as a function of the time of electrolysis. Dots are related to the oligo-(*S*)-BT_2_T_4_/Ppy hybrid tube, while triangles to the oligo-(*R*)-BT_2_T_4_/Ppy one.

For a tube with an oligo-(*S*)-BT_2_T_4_ layer, the chromatograms show a gradual and systematic decrease in the AP peak and a corresponding increase in the (*S*)-PE peak over time (Figure 2(A)). A specular result was obtained with the oligo-(*R*)-BT_2_T_4_ tube (Figure S10). A quantitative analysis (Figure 2(B)) reveals that the product yield increases steeply over the first 30 min to ∼ 80%, eventually reaching a near-quantitative conversion of 99% after 60 min. Remarkably, this dramatic enhancement in yield was achieved without compromising stereocontrol; the ee remained high, above 94%, throughout the entire process. Reproducibility experiments confirmed these findings, with multiple runs consistently delivering yields over 95% and ee values over 94% (Figure S11). This demonstrates that the AC method is a highly effective and robust strategy for achieving both high conversion and enantioselectivity. We further explored the tunability of the AC system by varying the applied electric field and frequency. First, experiments were conducted at three different field strengths: 1.2, 1.4, and 1.6 V/cm. As predicted by BE theory, a stronger electric field increases the length of the polarized zones at the ends of the BPE.

As shown in Figure 3, increasing the field strength significantly accelerated the reaction rate. At 1.6 V/cm, 99% conversion was reached in just 40 min, whereas at 1.2 V/cm, it took 80 min to achieve the same yield. This is because a larger portion of the tube’s inner surface is at a sufficient potential for reduction at higher fields (Figure S5). Despite the variation in kinetics, the enantioselectivity remained excellent (>90% ee) in all cases, highlighting the system’s tunability. Interestingly, at the highest field (1.6 V/cm), the yield began to decrease after reaching its maximum at 40 min (Figure 3(B)).

**Figure 3:**
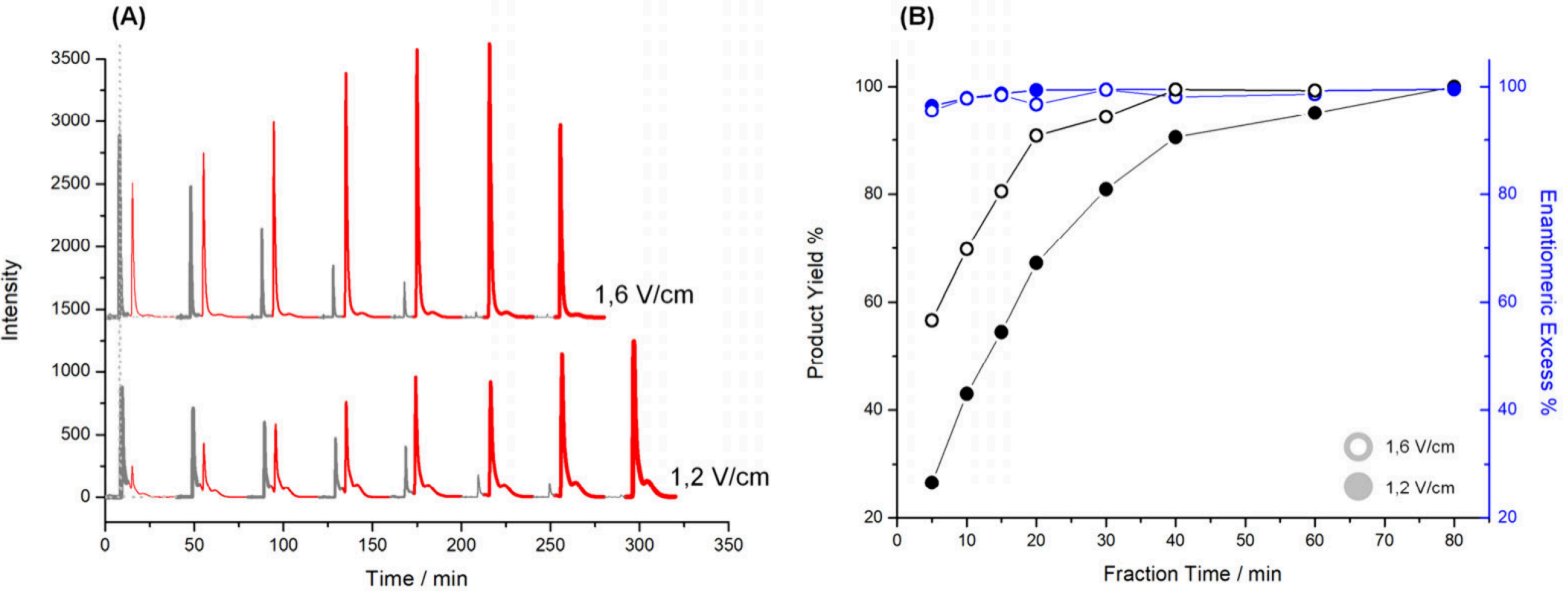
Results related to the alternating current asymmetric electrosynthesis at constant electric fields of 1.2 V/cm and 1.4 V/cm. (A) Chromatograms of the fractions collected at different reaction times (5, 10, 15, 20, 30, 40, 60 min, as well as 80 min for the 1.2 V/cm case) from the δ^+^ extremity of oligo-(*S*)-BT_2_T_4_/Ppy tubes. The red color stands for (*S*)-PE, while the gray peaks are related to the AP. For comparison, the gray dotted line represents the AP prior to injection into the hybrid tube. (B) Enantiomeric excess (in blue) and product yield (in black) as a function of electrolysis time. Solid dots correspond to the 1.2 V/cm electric field, while empty dots represent the 1.6 V/cm one.

This is attributed to the back-oxidation of the product (PE) to the starting material (AP) at the anodic pole. Once all the AP is consumed, the PE produced at the cathode is shuttled to the anode upon field inversion, where it can be oxidized, thus lowering the overall yield. This effect is not seen at lower fields because the reaction is slower and does not reach full conversion within the same time frame. Specular results were also obtained with the oligo-(*R*)-BT_2_T_4_ tube for the lowest and highest electric fields applied (Figure S12). Second, the effect of AC frequency (*f*) was investigated using a 0.5 cm tube at a constant field of 2.8 V/cm. The reaction was run for 30 min at frequencies of 0.006, 0.011, and 0.022 Hz.

The results (Figure S13) show a strong dependence of yield on frequency. At the lowest frequency (0.006 Hz, corresponding to a long period), the yield was 94%, while at the highest frequency (0.022 Hz), it was only 13%.

This is attributed to the efficiency of fluid mixing. At low frequencies, the slow ″back-and-forth″ motion of the fluid ensures that the entire sample is well-mixed and repeatedly contacts the active surface. At high frequencies, the rapid switching of polarity leads to a pseudostationary state where the fluid barely moves, resembling a batch reactor with poor mass transport and thus low conversion. Again, the enantioselectivity remained high (>90% *ee*) for all frequencies, underscoring the robustness of the chiral recognition mechanism. To provide a more rigorous, quantitative explanation for the frequency dependence shown in Figure S13, we analyze the system in the context of mass transport in oscillatory flows.^53,54^ The nature of the oscillatory flow is described by the Womersley number, $\alpha =R\sqrt{\omega }/\vartheta$ , where R is the tube radius, ω is the angular frequency, and ϑ is the kinematic viscosity.^55^ For all tested frequencies (0.006–0.022 Hz), the Womersley number remains very small (α≪1), indicating that the flow is always in a viscosity-dominated (quasi-steady) regime where the entire fluid slug oscillates with a parabolic velocity profile. The key factor governing the overall conversion is not the reaction kinetics (which are constant), but the efficiency of mass transport, which dictates the reactor’s Residence Time Distribution (RTD).^56^ The AC frequency acts as a control parameter to tune the dominant mode of mass transport, shifting the reactor’s behavior between two well-defined limits. The interplay between the parabolic shear flow and transverse diffusion gives rise to an effective axial dispersion, a phenomenon known as Taylor-Aris dispersion.^57,58^ High axial dispersion leads to a broad RTD, characteristic of a continuous stirred tank reactor (CSTR), whereas low axial dispersion leads to a narrow RTD that approaches ideal plug-flow reactor (PFR) behavior. At low frequencies (e.g., 0.006 Hz), the long oscillation period allows for large-amplitude convective shuttling of the entire reactant droplet. This large-scale mixing dominates over axial dispersion, effectively averaging the experience of all fluid elements and ensuring they are repeatedly exposed to the catalytic wall. This regime approximates a PFR with a very long effective residence time, characterized by a narrow RTD. Consequently, nearly all reactant molecules have sufficient time to react, resulting in the observed high conversion (94%). At high frequencies (e.g., 0.022 Hz), the oscillation amplitude becomes minimal. Convective mixing is suppressed, and the system becomes dominated by Taylor-Aris dispersion. The pronounced velocity shear, coupled with slow radial diffusion, leads to a high effective axial dispersion coefficient (*D*_eff_) and thus a very broad RTD. In this regime, fluid elements in the center of the tube have a very short residence time in the active zones and effectively bypass the catalyst, while only a small fraction of the fluid near the wall reacts. This behavior is analogous to a poorly mixed reactor with significant channeling, explaining the sharp drop in overall conversion to just 13%.

### 2.5 Expanding to Wireless Enantioselective Oxidation

To demonstrate the versatility of the reactor design in terms of application, it has been also used for an enantioselective oxidation reaction: the conversion of prochiral lansoprazole sulfide (Lans-S) to the pharmaceutically important proton-pump inhibitor lansoprazole (Lans), which exists as d- and l-enantiomers. Conventional enantioselective tests confirmed that the oligo-(*R*)-BT_2_T_4_ material preferentially interacts with d-lansoprazole (Figure S9A). The experiment was conducted at 1.6 V/cm using an oligo-(*R*)-BT_2_T_4_ tube to target the synthesis of d-lansoprazole (d-lans).

The Lans-S was dissolved in a small amount of an ionic liquid to facilitate its introduction into the aqueous system. Fractions were collected after 45 and 90 min. As shown in Figure 4, the system successfully produced d-lans with excellent enantioselectivity (>90% ee). The reaction was slower than the AP reduction, reaching a yield of 85% after 90 min.

**Figure 4:**
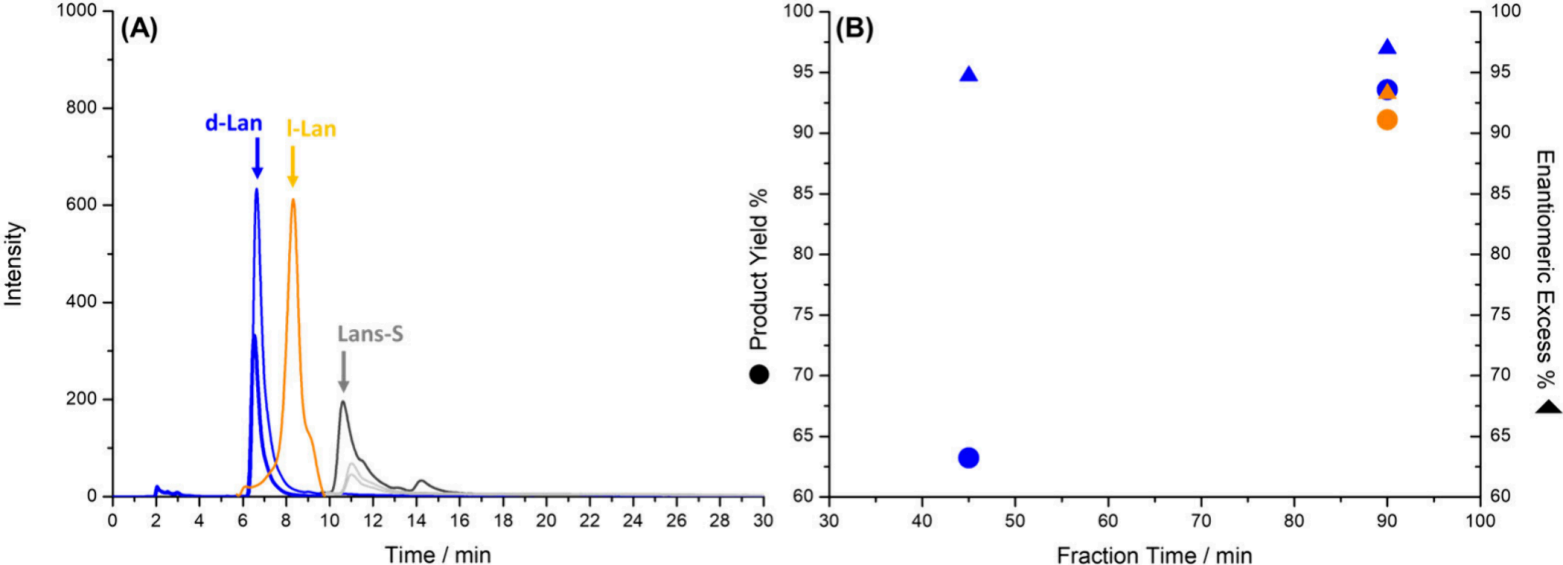
Results related to the alternating current asymmetric electrooxidation of Lans-S at a constant electric field of 1.6 V/cm. (A) Chromatograms of the fractions collected from the δ^–^ extremity of oligo-(*R*)- (after 45 and 90 min of reaction) or oligo-(*S*)-BT_2_T_4_/Ppy (after 90 min) tubes, by applying a constant electric field of 1.6 V/cm. The blue and orange colors stand for d- and l-lans, respectively, while the gray peaks are related to lans-S. (B) Enantiomeric excess (triangles) and product yield (dots) as a function of reaction time. Blue dots correspond to the d-lans, while the orange ones represent the l-lans.

This highlights the tunability of the system, where reaction parameters can be optimized for different substrates and transformations. Using the oligo-(*S*)-BT_2_T_4_ tube under the same conditions resulted in the selective formation of l-lans, further confirming the predictable stereocontrol of the reactor. The long-term stability of the reactor is a critical parameter for practical applications. We investigated the reusability of a single tube over multiple 1 h AC synthesis cycles. The device maintained over 95% of its initial efficiency for two consecutive cycles. However, during the third cycle, a significant drop in both pumping action and reaction yield (to ∼ 65% of the initial value) was observed. To understand this degradation, post-mortem analysis was performed on the used tubes. Electrochemical Impedance Spectroscopy (EIS) revealed a significant increase in the charge-transfer resistance (*R*_ct_) compared to a pristine device (Table S2). This finding is consistent with the known degradation pathway of Ppy under prolonged or excessive anodic polarization.^59^ The mechanism involves the irreversible overoxidation of the Ppy backbone, where nucleophilic species in the electrolyte attack the positively charged polymer chain, leading to a loss of π-conjugation. This degradation compromises the polymer’s electrical conductivity and, consequently, its ability to undergo the volume changes necessary for electromechanical actuation. In contrast, CV analysis of the inner oligo-BT_2_T_4_ layer showed only a minor loss of electrochemical activity. This confirms that the primary failure mode is the degradation of the outer Ppy actuator layer, not the inner chiral catalyst.

This knowledge is crucial for future material design, suggesting that more robust conducting polymers, such as Ppy-graphene composites, could significantly enhance the operational lifetime of the device.

### 2.6 Further Broadening the Synthetic Utility with Asymmetric Reductive Amination

Having established and rigorously validated a predictive, atomic-level model for the stereocontrol exerted by the oligo-BT_2_T_4_ catalyst, its robustness has been challenged with a synthetically demanding transformation that would simultaneously close the loop of the mechanistic investigations. To this end, the reactor’s capability was tested for the asymmetric reductive amination of acetylferrocene^60^ with dimethylamine to directly synthesize (*R*)-N,N-dimethyl-1-ferrocenylethylamine, (*R*)-Fc, the chiral ferrocene probe, historically known as Ugi’s amine,^61^ that was central to the preceding host–guest binding studies.

This choice of target is a deliberate and powerful demonstration of the system’s broad utility. The ferrocene derivative, initially employed as a diagnostic tool to establish the fundamental principle of chiral recognition, is now proposed as an integral component of a high-value synthetic target. This transition from mechanistic probe to final product provides the ultimate validation of the model, demonstrating that the principles of stereorecognition elucidated with the probe are directly and effectively translated into a practical catalytic application.

The reaction, carried out with the optimized AC protocol at an electric field of 1.4 V/cm and the oligo-(*R*)-BT_2_T_4_/Ppy hybrid tube, proceeded smoothly to afford the desired (*R*)-Fc in 89% isolated yield and with an enantiomeric excess of >99.5%, as determined by chiral HPLC analysis (Figure 5).^29,30^

**Figure 5:**
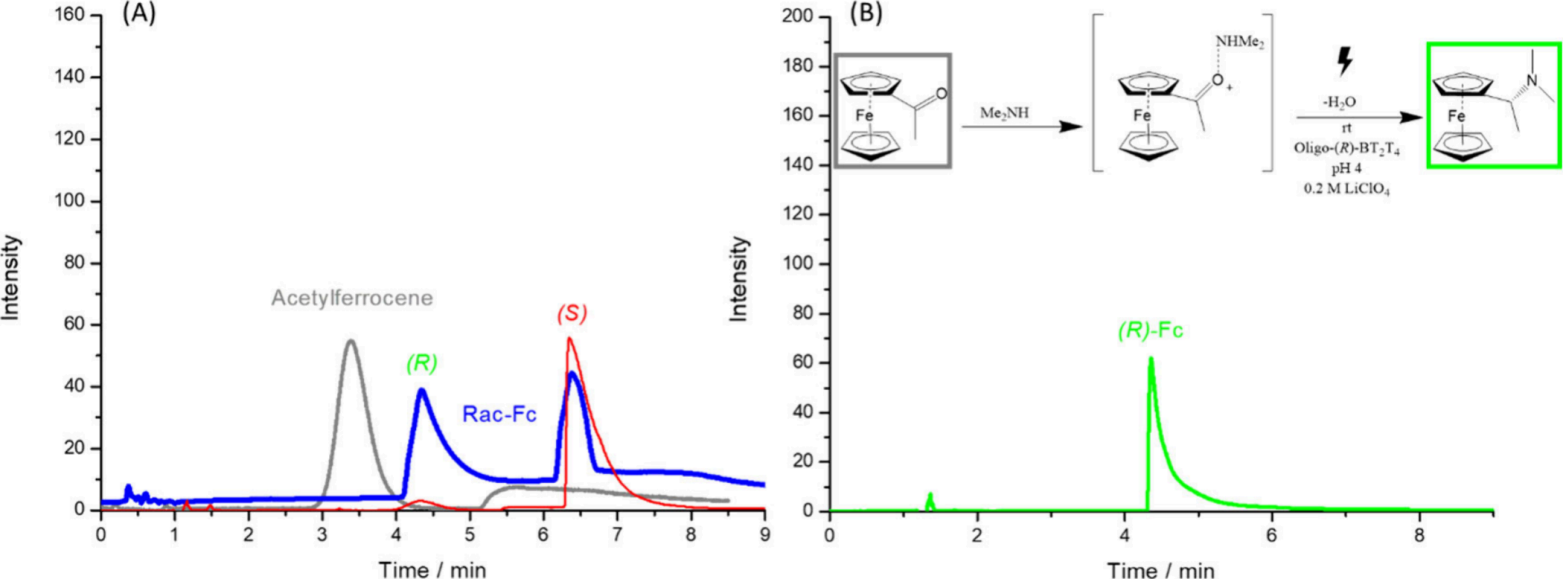
(A) Chiral HPLC chromatograms of pristine acetylferrocene in gray, Rac-Fc in blue and (*S*)-Fc in red. (B) Reaction scheme for the reductive amination of acetylferrocene and dimethylamine to form (*R*)-N,N-dimethyl-1-ferrocenylethylamine. Chiral HPLC chromatogram of the product obtained using an oligo-(*R*)-BT_2_T_4_/Ppy hybrid tube, showing the selective formation of a single enantiomer.

This high level of stereocontrol is not only remarkable for an electrochemical reductive amination,^62^ but also stands as a compelling confirmation of the dual-control stereochemical model.

The model, validated by both DFT calculations and KIE experiments for acetophenone reduction, indicates that selectivity arises from a combination of steric repulsion of the substrate’s bulky group and stabilizing C–H···π interactions involving its methyl group. The acetylferrocene substrate is, in indeed, a perfect test case for this model. The sterically demanding ferrocenyl group, which is significantly larger than the phenyl group of acetophenone, is predicted to experience an even greater destabilizing steric clash with the catalyst’s backbone in the unfavorable transition state.

This amplified steric repulsion increases the energetic penalty for the undesired pathway. Concurrently, the substrate’s methyl group remains perfectly positioned to engage in the stabilizing C–H···π interaction with the oligomer’s thiophene rings, a key interaction whose kinetic relevance was established by the KIE studies. The combination of these two effects, steric hindrance for the unfavorable pathway and preserved stabilization for the favored one, results in a larger differential activation energy and enantioselectivity (ΔΔG^‡^= +17.1 kJ/mol).

## 3 Conclusion

In this work, we have designed and successfully operated an all-in-one, self-pumping bipolar electrochemical reactor for asymmetric synthesis. By synergistically integrating the electromechanical actuation of a Ppy outer layer with the outstanding enantioselective properties of an inner “inherently chiral” oligomer film, we have developed a miniaturized flow system that operates wirelessly without external pumps. A key innovation is the implementation of an AC-driven protocol that boosts reaction yield to high levels (99%) while maintaining high stereoselectivity (>99% ee).

The system’s utility has been demonstrated via three mechanistically distinct transformations, ketone reduction, sulfide oxidation, and reductive amination, culminating in the direct asymmetric synthesis of Ugi’s amine with elevated stereocontrol (>99.5% ee), which closes the loop from mechanistic probe to synthetic product.

Through a combination of fundamental kinetic investigations (Eyring analysis and Kinetic Isotope Effect studies) and state-of-the-art Density Functional Theory (DFT) calculations, we have developed an experimentally validated, predictive model for enantioselection.

The successful synthesis of Ugi’s amine, a result that demonstrates the predictive power of the model even for a sterically demanding substrate, shows that the computational protocol has the potential to be a valuable tool for *in silico* catalyst design. This study thus lays the foundation for a rational, computationally guided design of smart, automated systems for highly efficient wireless asymmetric electrosynthesis.

## Supplementary Material







## Notes

Macroscopic images of the oligo-BT_2_T_4_/Ppy hybrid tube, cyclic voltammetry data, chromatograms from HPLC analyses, theoretical polarization potential profiles, ssNMR, EQCM and MCD experiments, schematic representation of the alternating current electrosynthesis method, intra-tubular flow quantification, theoretical framework for electro-chemo-mechanical pumping, Eyring plot, enantiomeric excess and conversion yield equations, DFT calculations, KIE studies, experimental procedures (PDF)
“Forward-and-backward” shuttling of the reactant droplet (AVI)
Alternating current-driven liquid flow (AVI)

## Abbreviations

AC: Alternating Current
ACN: Acetonitrile
AP: Acetophenone
BE: Bipolar Electrochemistry
BPE: Bipolar Electrode
BT2T4: 2,2′-bis­[2-(5,2′-bithienyl)]-3,3′-bithianaphthene
CV: Cyclic Voltammetry
DC: Direct Current
ee: Enantiomeric Excess
HPLC: High-Performance Liquid Chromatography
Hex: Hexane
IPA: Isopropanol
LiClO_4_: Lithium Perchlorate
Lans: Lansoprazole
Lans-S: Lansoprazole Sulfide
Ppy: Polypyrrole
PE: 1-Phenylethanol
ssNMR: Solid State Nuclear Magnetic Resonance
MAS: magic angle spinning
cpmas: Cross-Polarization
hpdec: high-power proton decoupling
EQCM: Electrochemical Quartz Crystal Microbalance
MCD: Magnetic Circular Dichroism.

## References

[1] Smith S. W. (2009). Chiral
toxicology: It’s the same thing···only different. Toxicol. Sci..

[2] Calcaterra A., D’Acquarica I. (2018). The market of chiral drugs: Chiral switches versus
de novo enantiomerically pure compounds. J.
Pharm. Biomed Anal..

[3] Liu W., Gan J., Schlenk D., Jury W. A. (2005). Enantioselectivity
in environmental
safety of current chiral insecticides. Proc.
Natl. Acad. Sci. U.S.A..

[4] Alvarez-Rivera G., Bueno M., Ballesteros-Vivas D., Cifuentes A. (2020). Chiral analysis in
food science. TrAC.

[5] Wang H., Li M.-H., Liu H., Wang Y.-L., Zhu J.-W., Lu J.-X. (2024). Recent advances
in chiral electrodes for asymmetric electrosynthesis. ChemCatChem..

[6] Yamamoto K., Kuriyama M., Onomura O. (2021). Asymmetric electrosynthesis: recent
advances in catalytic transformations. Curr.
Opin. Electrochem..

[7] Qian H.-L., Xu S.-T., Yan X.-P. (2023). Recent advances in separation and
analysis of chiral compounds. Anal. Chem..

[8] Cheng Q., Ma Q., Pei H., He S., Wang R., Guo R., Liu N., Mo Z. (2023). Enantioseparation
membranes: research status, challenges,
and trends. Small.

[9] Malacarne F., Grecchi S., Niamlaem M., Bonczak B., Salinas G., Arnaboldi S. (2024). Unconventional approaches for chiral resolution. Anal Bioanal Chem..

[10] Fanourakis A., Docherty P. J., Chuentragool P., Phipps R. J. (2020). Recent developments
in enantioselective transition metal catalysis featuring attractive
noncovalent interactions between ligand and substrate. ACS Catal..

[11] Pellissier H. (2017). Recent developments
in enantioselective lanthanide-catalyzed transformations. Coord. Chem. Rev..

[12] Wagen C. C., McMinn S. E., Kwan E. E., Jacobsen E. N. (2022). Screening
for generality
in asymmetric catalysis. Nature.

[13] Xia Q.-H., Ge H.-Q., Ye C.-P., Liu Z.-M., Su K.-X. (2005). Advances
in homogeneous and heterogeneous catalytic asymmetric epoxidation. Chem. Rev..

[14] Heitbaum M., Glorius F., Escher I. (2006). Asymmetric heterogeneous catalysis. Angew. Chemie Int. Ed..

[15] Lemaire M. (2004). Heterogeneous
asymmetric catalysis. Pure Appl. Chem..

[16] Frontana-Uribe B. A., Little R. D., Ibanez J. G., Palma A., Vasquez-Medrano R. (2010). Organic electrosynthesis:
a promising green methodology in organic chemistry. Green Chem..

[17] Beil S. B., Pollok D., Waldvogel S. R. (2021). Reproducibility in electroorganic
synthesis—myths and misunderstandings. Angew. Chem., Int. Ed..

[18] Little R. D. (2020). A Perspective
on organic electrochemistry. J. Org. Chem..

[19] Pollok D., Waldvogel S. R. (2020). Electro-organic synthesis – a 21st century technique. Chem. Sci..

[20] Gellman A. J. (2010). Chiral
surfaces: accomplishments and challenges. ACS
Nano.

[21] Dybtsev D. N., Bryliakov K. P. (2021). Asymmetric
catalysis using metal-organic frameworks. Coord.
Chem. Rev..

[22] Mallat T., Orglmeister E., Baiker A. (2007). Asymmetric catalysis at chiral metal
surfaces. Chem. Rev..

[23] Somsri S., Suwankaisorn B., Yomthong K., Srisuwanno W., Klinyod S., Kuhn A., Wattanakit C. (2023). Highly enantioselective
synthesis of pharmaceuticals at chiral-encoded metal surfaces. Chem.—Eur. J..

[24] Fosdick S. E., Knust K. N., Scida K., Crooks R. M. (2013). Bipolar electrochemistry. Angew. Chem., Int. Ed..

[25] Karimian N., Hashemi P., Afkhami A., Bagheri H. (2019). The principles of bipolar
electrochemistry and its electroanalysis applications. Curr. Opin. Electrochem..

[26] Koefoed L., Pedersen S. U., Daasbjerg K. (2017). Bipolar electrochemistry—A
wireless approach for electrode reactions. Curr.
Opin. Electrochem..

[27] Rahn K. L., Anand R. K. (2021). Recent advancements in bipolar electrochemical
methods
of analysis. Anal. Chem..

[28] Wang Y.-L., Cao J.-T., Liu Y.-M. (2022). Bipolar
electrochemistry –
A powerful tool for micro/nano-electrochemistry. ChemistryOpen.

[29] Grecchi S., Salinas G., Cirilli R., Benincori T., Ghirardi S., Kuhn A., Arnaboldi S. (2024). Miniaturized
enantioselective tubular devices for the electromechanical wireless
separation of chiral analytes. Chem..

[30] Grecchi S., Malacarne F., Cirilli R., Dell’Edera M., Ghirardi S., Benincori T., Arnaboldi S. (2024). Wireless hollow
miniaturized objects for electroassisted chiral resolution. Anal. Chem..

[31] Sannicolò F., Arnaboldi S., Benincori T., Bonometti V., Cirilli R., Dunsch L., Kutner W., Longhi G., Mussini P. R., Panigati M., Pierini M., Rizzo S. (2014). Potential-driven
chirality manifestations and impressive enantioselectivity by inherently
chiral electroactive organic films. Angew. Chem.,
Int. Ed..

[32] Arnaboldi S., Grecchi S., Magni M., Mussini P. R. (2018). Electroactive chiral
oligo and polymer layers for electrochemical enantiorecognition. Curr. Opin. Electrochem..

[33] Rosetti A., Bonetti G., Villani C., Benincori T., Cirilli R. (2021). Multimilligram-scale production implementation
of atropisomers
of 2,2′-bis­(2,2′-bithiophene-5-yl)-3,3′-bithianaphthene. Chirality..

[34] Gupta B., Zhang L., Melvin A. A., Goudeau B., Bouffier L., Kuhn A. (2021). Designing tubular conducting polymer actuators for wireless electropumping. Chem. Sci..

[35] Khan A., Alamry K. A., Jain R. K. (2019). Polypyrrole nanoparticles-based soft
actuator for artificial muscle applications. RSC Adv..

[36] Wang X., Li L., Liu E., Wang J., Han X., Cao Y., Lu C. (2021). High-performance
multiresponsive bilayer actuators based on micro-/nanostructured
polypyrrole for robust smart devices. ACS Appl.
Nano Mater..

[37] Wang T., Li M., Zhang H., Sun Y., Dong B. (2018). A multi-responsive
bidirectional bending actuator based on polypyrrole and agar nanocomposites. J. Mater. Chem. C.

[38] Tyagi M., Fathollahzadeh M., Martinez J. G., Mak W. C., Filippini D., Jager E. W. (2023). Radially actuating conducting polymer microactuators
as gates for dynamic microparticle sieve based on printed microfluidics. Sens. Actuators, B.

[39] Pramanik S. K., Suzuki H. (2020). Switchable microvalves employing a conducting polymer
and their automatic operation in conjunction with micropumps with
a superabsorbent polymer. ACS Appl. Mater. Interfaces.

[40] Gupta B., Goudeau B., Kuhn A. (2017). Wireless Electrochemical
Actuation
of Conducting Polymers. Angew. Chem., Int. Ed..

[41] Zhong Y., Filippini D., Jager E. W. H. (2021). A versatile flexible polymer actuator
system for pumps, valves, and injectors enabling fully disposable
active microfluidics. Adv. Materials Technologies.

[42] Sunoj R. B. (2016). Transition
State Models for Understanding the Origin of Chiral Induction in Asymmetric
Catalysis. Acc. Chem. Res..

[43] Cusumano A. Q., Stoltz B. M., Goddard W. A. (2020). Reaction Mechanism,
Origins of Enantioselectivity, and Reactivity Trends in Asymmetric
Allylic Alkylation: A Comprehensive Quantum Mechanics Investigation
of a C­(sp^3^)–C­(sp^3^) Cross-Coupling. J. Am. Chem. Soc..

[44] Holder J. C., Zou L., Marziale A. N., Liu P., Lan Y., Gatti M., Kikushima K., Houk K. N., Stoltz B. M. (2013). Mechanism and Enantioselectivity
in Palladium-Catalyzed Conjugate Addition of Arylboronic Acids to
β-Substituted Cyclic Enones: Insights from Computation and Experiment. J. Am. Chem. Soc..

[45] Maloney M. P., Stenfors B. A., Helquist P., Norrby P.-O., Wiest O. (2023). Interplay
of Computation and Experiment in Enantioselective Catalysis: Rationalization,
Prediction, and Correction?. ACS Catal..

[46] Kanie O., Shioiri Y., Ogata K., Uchida W., Daikoku S., Suzuki K., Nakamura S., Ito Y. (2016). Diastereomeric resolution
directed towards chirality determination focussing on gas-phase energetics
of coordinated sodium dissociation. Sci. Rep..

[47] Matysik S. C., Wales D. J., Jenkins S. J. (2023). Dynamic
Diastereomerism on Chiral
Surfaces. J. Phys. Chem. C.

[48] Biosca, M. ; Besora, M. ; Maseras, F. ; Pàmies, O. ; Diéguez, M. Chapter Two-Combining DFT and experimental studies in enantioselective catalysis: From rationalization to prediction. In Advances in Catalysis; Elsevier, 2024; Vol. 75, pp 23–54.

[49] Sannicolò F., Rizzo S., Benincori T., Kutner W., Noworyta K., Sobczak J. W., Bonometti V., Falciola L., Mussini P., Pierini M. (2010). An effective multipurpose building block for 3D electropolymerisation:
2,2′-Bis­(2,2′-bithiophene-5-yl)-3,3′-bithianaphthene. Electrochim. Acta.

[50] Niamlaem M., Grecchi S., Matthayom P., Warakulwit C., Maggioni D., Arnaboldi S. (2025). Unlocking the power of chirality:
Surface nanoarchitectonics of modified halloysite nanotubes for enantioselective
recognition. Talanta.

[51] Anslyn, E. V. ; Dougherty, D. A. Modern Physical Organic Chemistry; University Science Books, 2006.

[52] Singleton D. A., Thomas A. A. (1995). High-Precision
Simultaneous Determination of Multiple
Small Kinetic Isotope Effects at Natural Abundance. J. Am. Chem. Soc..

[53] Watson E. J. (1983). Diffusion
in oscillatory pipe flow. J. Fluid Mech..

[54] Chatwin P. C. (1975). On the
longitudinal dispersion of passive contaminant in oscillatory flows
in tubes. J. Fluid Mech..

[55] Womersley J. R. (1955). Method
for the calculation of velocity, rate of flow and viscous drag in
arteries when the pressure gradient is known. J. Physiol..

[56] Danckwerts P. V. (1953). Continuous
flow systems: Distribution of residence times. Chem. Eng. Sci..

[57] Taylor G. I. (1953). Dispersion
of soluble matter in solvent flowing slowly through a tube. Proc. R. Soc. London A.

[58] Aris R. (1956). On the dispersion
of a solute in a fluid flowing through a tube. Proc. R. Soc. London A.

[59] Marchesi L. F. Q. P., Simoes F. R., Pocrifka L. A., Pereira E. C. (2011). Investigation of
polypyrrole degradation using electrochemical impedance spectroscopy. J. Phys. Chem. B.

[60] Storer R.
I., Carrera D. E., Ni Y., MacMillan D. W. C. (2006). Enantioselective
Organocatalytic Reductive Amination. J. Am.
Chem. Soc..

[61] Zhu J.-C., Cui D.-X., Li Y.-D., Jiang R., Chen W.-P., Wang P.-A. (2018). ChemCatChem..

[62] Rooney C. L., Sun Q., Shang B., Wang H. (2025). Electrocatalytic Reductive Amination
of Aldehydes and Ketones with Aqueous Nitrite. J. Am. Chem. Soc..

