# Supplementary material for: Unplugging Asymmetric Synthesis with a Wireless, Self-Pumping Electrochemical Reactor
Source: J Am Chem Soc. 2025 Dec 10;147(51):47467–76. doi: 10.1021/jacs.5c16187 (PMC12752701; doi:10.1021/jacs.5c16187)
Supplement: Supplementary file 1 [file ja5c16187_si_001.pdf]

Supplemental Information

Unplugging Asymmetric Synthesis with a Wireless, Self-Pumping Electrochemical Reactor

Sara Grecchi,[1] Gerardo Salinas,[2] Malinee Niamlaem,[1] Alexander Kuhn,[2] Serena Arnaboldi[1*]

[1]Dipartimento di Chimica, Università degli Studi di Milano, Via Golgi 19, 20133 Milan, Italy

[2] Univ. Bordeaux, CNRS, Bordeaux INP, ISM, UMR 5255, 33607 Pessac, France

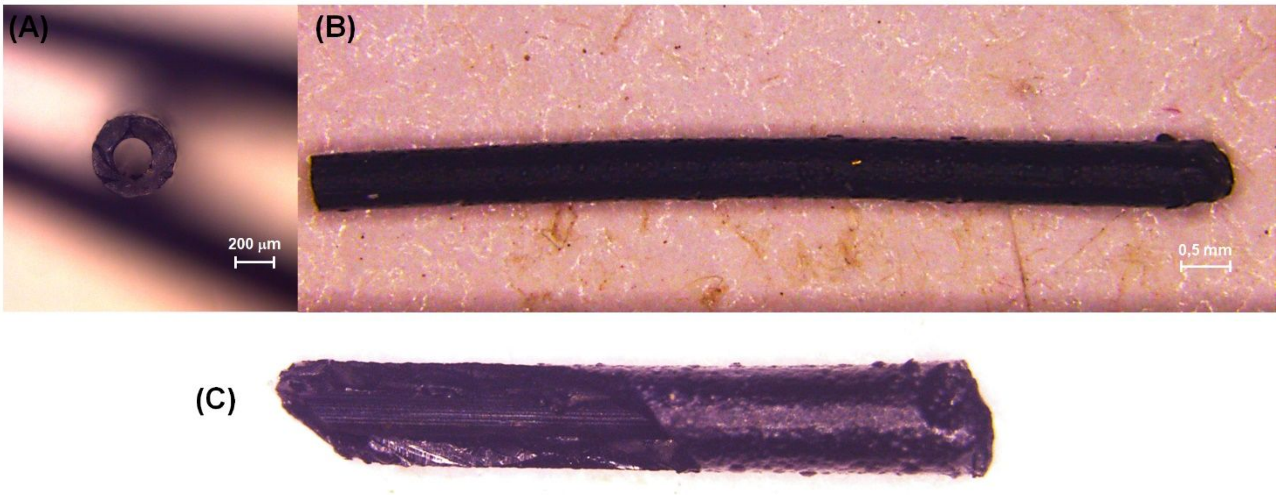

Figure S1. Macroscopic images of the oligo-BT_2T_4/Ppy hybrid tube. (A) Front view of the hollow tube. (B) Top view of the 1 cm-long tube and (C) Cross-sectional view showing the internal and external surfaces of the tube.

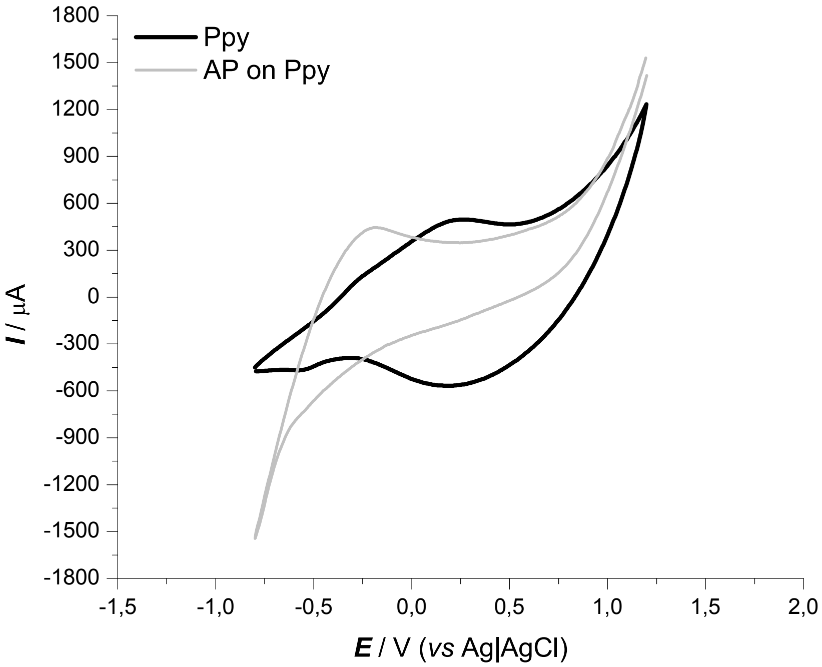

Figure S2. CV patterns of a self-standing Ppy strip in a pH 4 buffer + 0.2 M $LiClO_4$ solution in the absence (black line) and in the presence of 10 mM acetophenone (grey line).

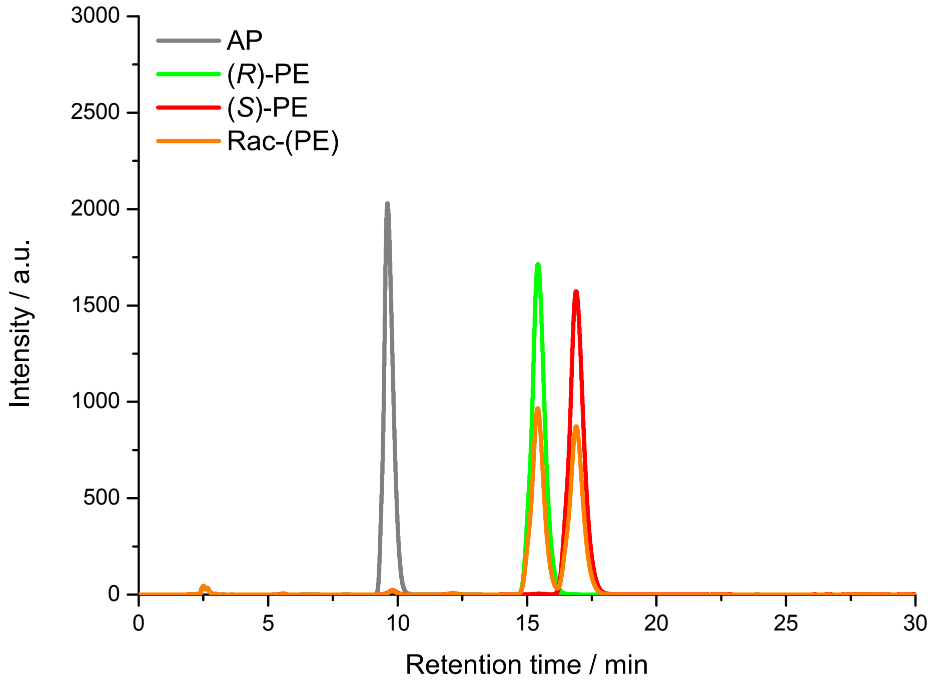

Figure S3. Chromatograms related to the direct HPLC analyses of pristine AP (in gray), (*R*)- and (*S*)-PE (in green and red, respectively) and racemic PE (in orange).

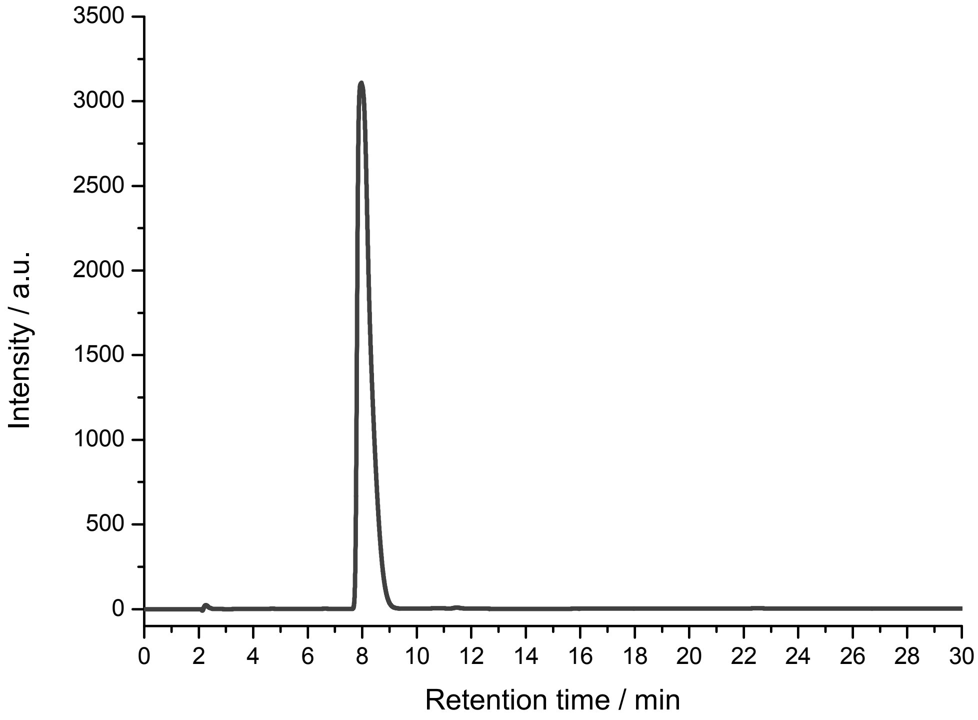

Figure S4. Chromatogram related to the 10 µL drop of AP before the BE experiment dissolved in 1.5 mL of heptane.

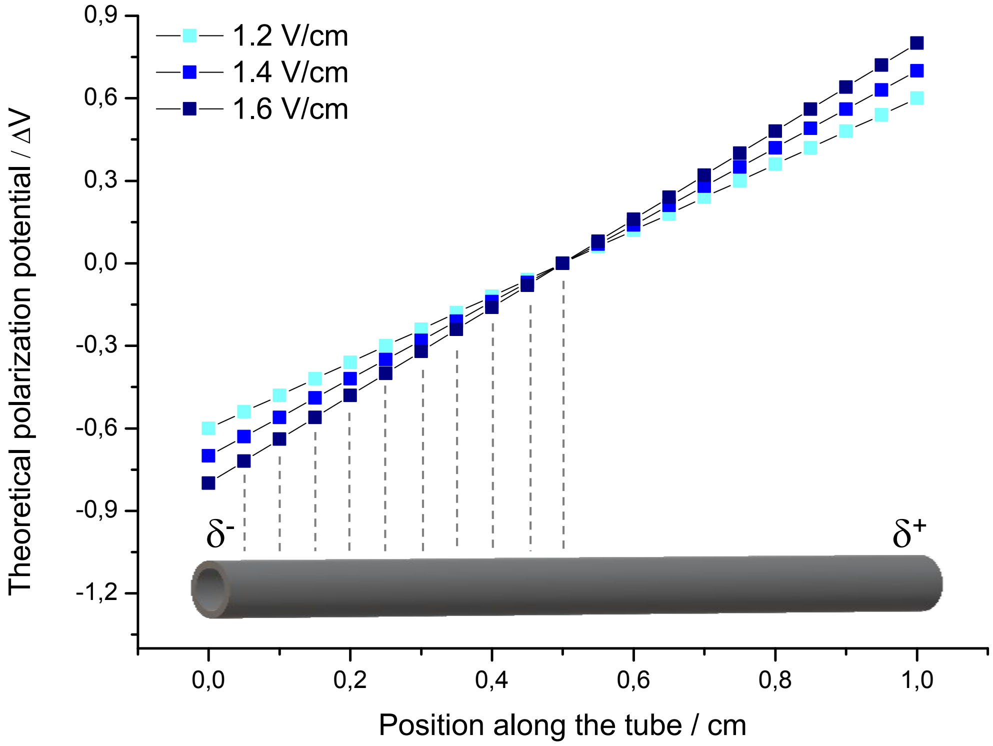

Figure S5. Theoretical polarization potential profiles as a function of the position along the 1 cm-long tube, under three different externally applied electric fields (1.2 V/cm in light blue, 1.4 V/cm in blue and 1.6 V/cm in dark blue).

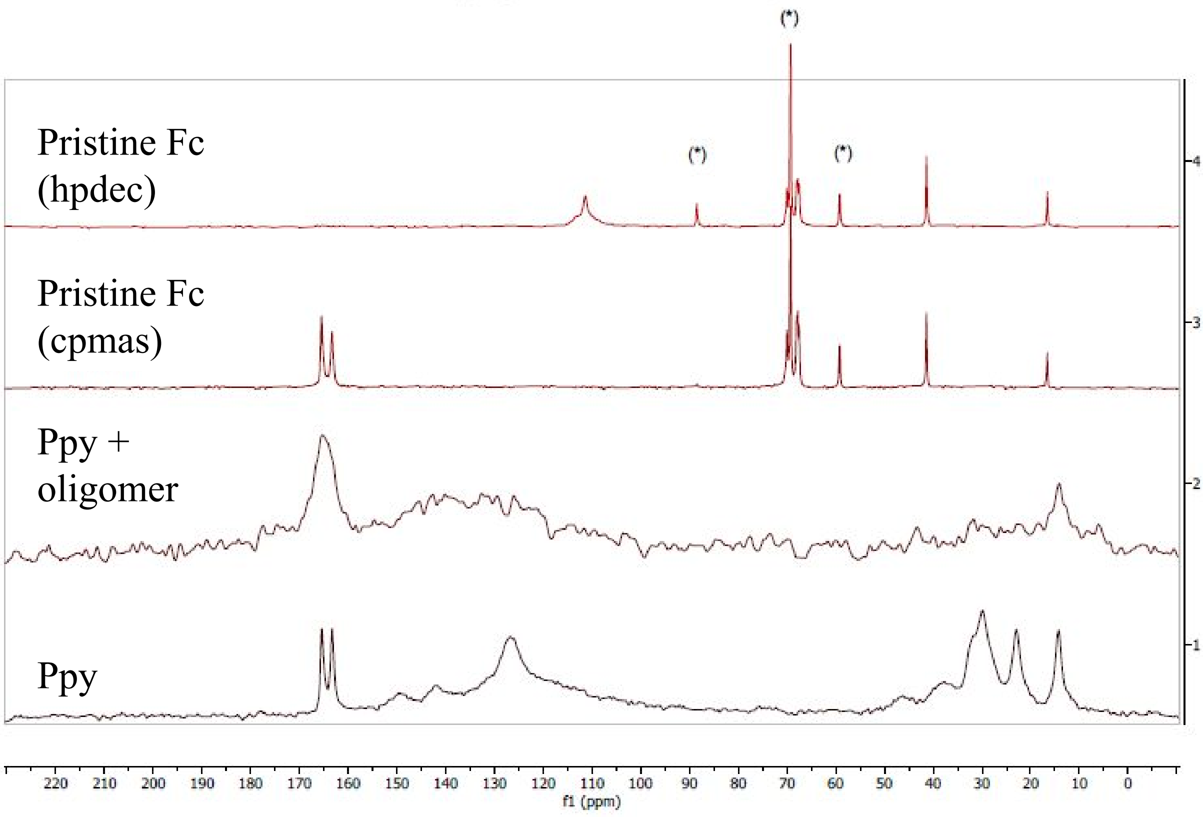

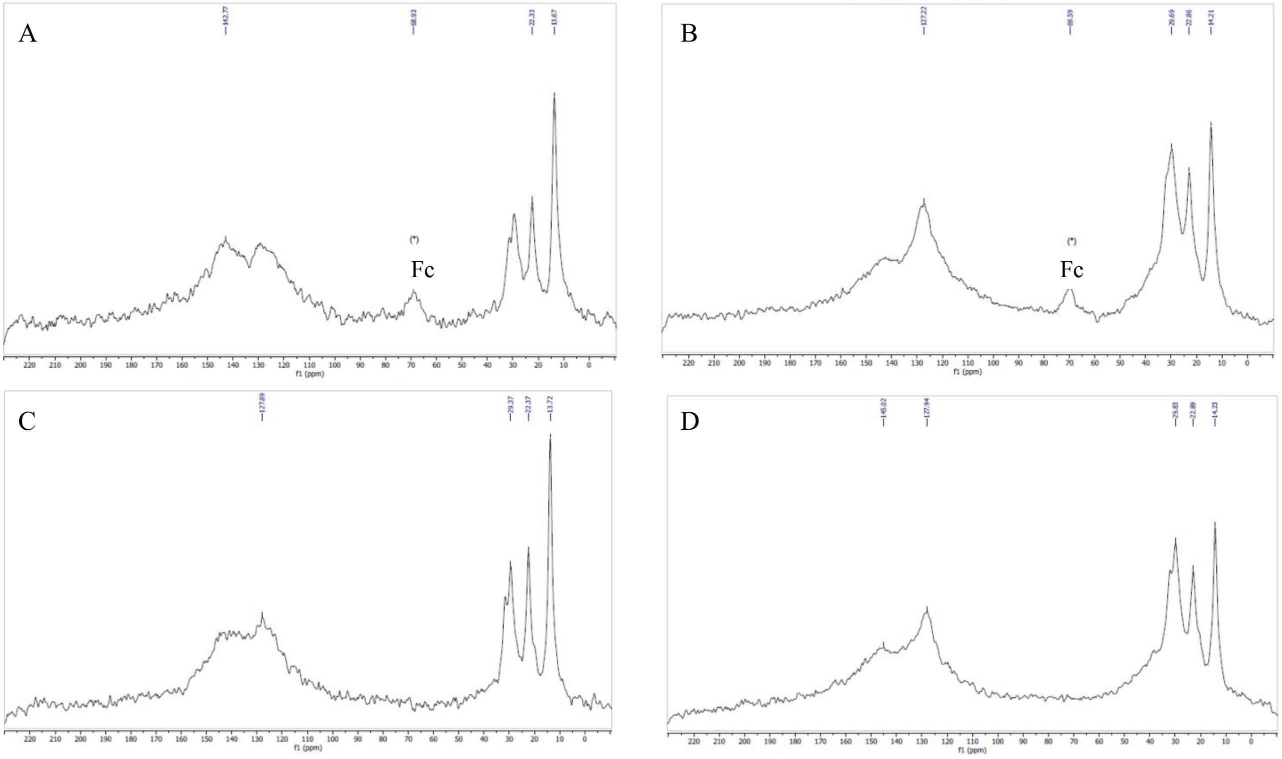

Figure S6. Top: ssNMR ^{13}C spectra of pristine chiral Fc (both cpmas and hpdec), PPy and Ppy/oligomer hybrid tube. Bottom: ssNMR ^{13}C spectra of the Ppy/oligo-*R* composite material after interaction (for two hours) with either (*R*)- (A-B) or (*S*)-Fc (C-D). A and C graphs show ssNMR ^{13}C cpmas, while B and D ssNMR ^{13}C hpdec.

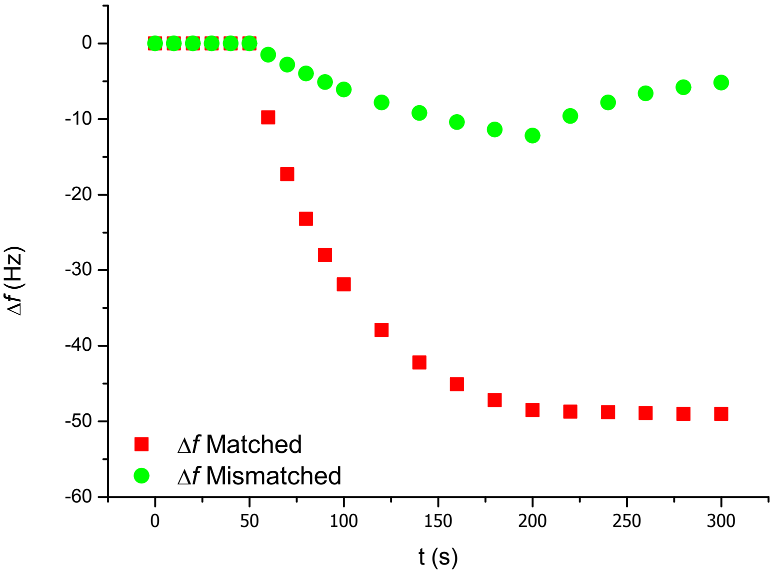

Figure S7. Preferential binding of (*R*)-Fc enantiomer to an (*R*)-oligomer-coated quartz electrode, quantified by EQCM. The frequency response demonstrates a significantly greater mass accumulation for the matching (*R*)-Fc (red squares) compared to the mismatching (*S*)-Fc (green squares). This differential response underscores the higher binding affinity of the matching pair ($|\Delta f_{matching}| > |\Delta f_{mismatching}|$).

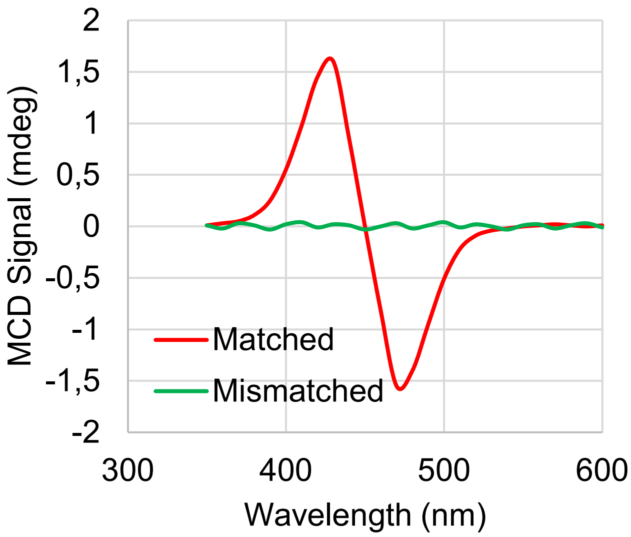

Figure S8. MCD spectra illustrate the distinct electronic signatures of the diastereomeric complexes formed between the chiral oligomer and the chiral ferrocene probe. The matching (*R*)-oligo/(*R*)-Fc complex exhibits a strong, derivative-shaped 'A-term' signal (red line), characteristic of a well-ordered, electronically perturbed ferrocene chromophore. In contrast, the non-binding mismatching enantiomer yields a negligible signal, indicating no significant electronic interaction (green line).

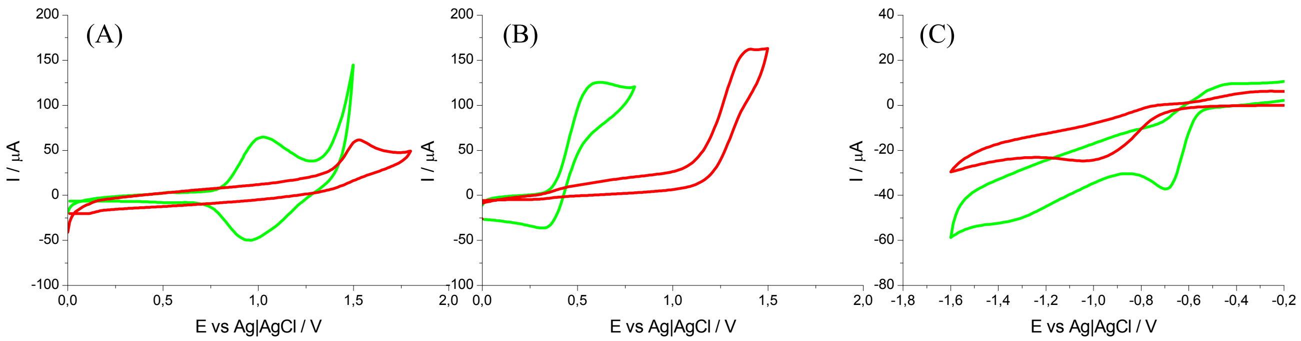

Figure S9. Enantioselective tests carried out in a 0.1 M $LiClO_4$ aqueous solution containing 2 mM (A) d-or l-lans, (B) (*R*)- or (*S*)- Fc and (C) (*R*)- or (*S*)-PE (in green and red, respectively) with an oligo-(*R*)-BT_2T_4 film, deposited on a graphite electrode.

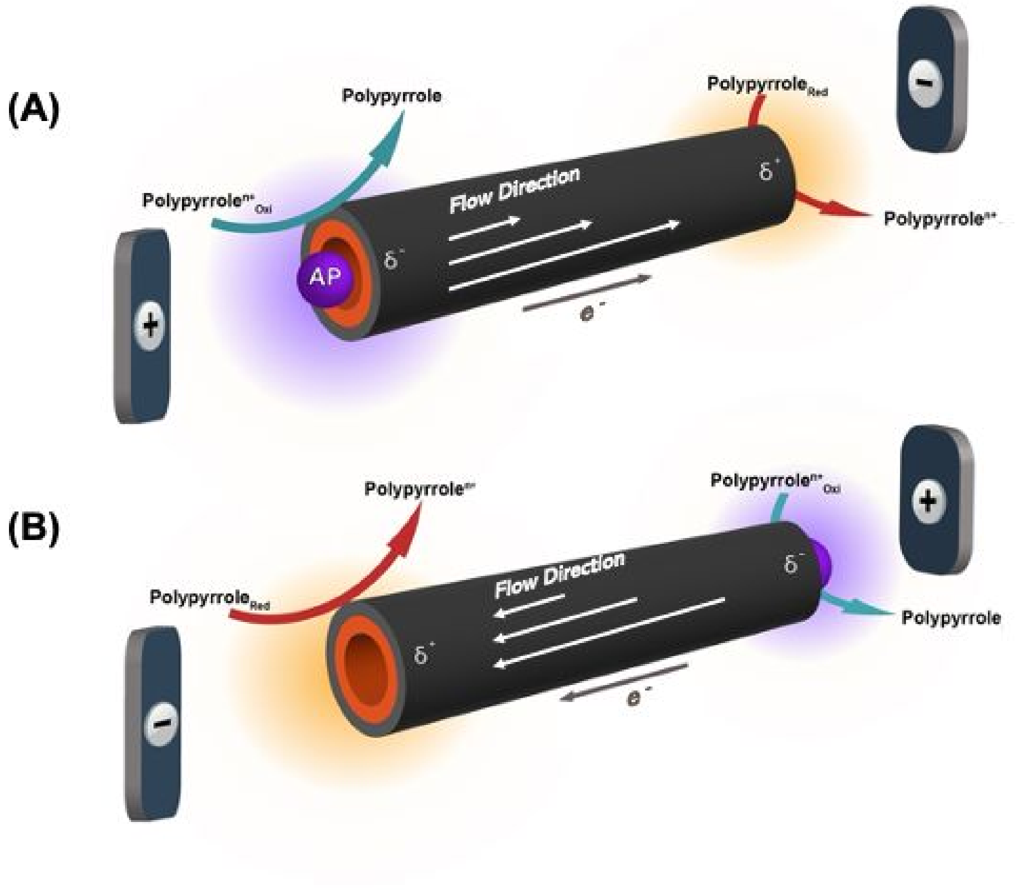

Scheme S1. Schematic representation of the alternating current electrosynthesis method, illustrating the "forward and backward" movement. The direction of ε and -ε, liquid flow, and the site of AP asymmetric reduction are shown. (A) Liquid flows from the anode to the cathode of the BPE, with AP reduction localized at the δ^- extremity. (B) Opposite electric field orientation, resulting in a specular electrochemical process, reversing the liquid flow direction and shifting the reduction to the new cathodic region.

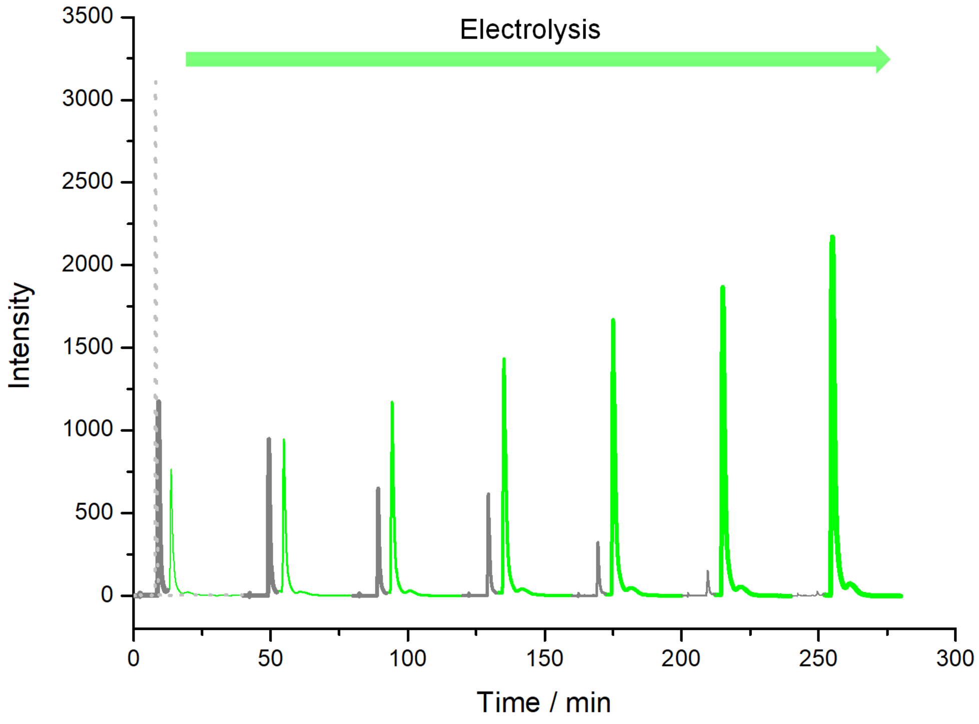

Figure S10. Chromatograms of the fractions collected at different reaction times (5, 10, 15, 20, 30, 40, 60 minutes) from the δ^+ extremity of an oligo-(*R*)-BT_2T_4/Ppy tube, by applying an electric field of 1.4 V/cm. The green colours stand for (*R*)-PE, while the grey peaks are related to AP. For comparison, the grey dotted line is associated with the AP concentration before the injection in the hybrid tube.

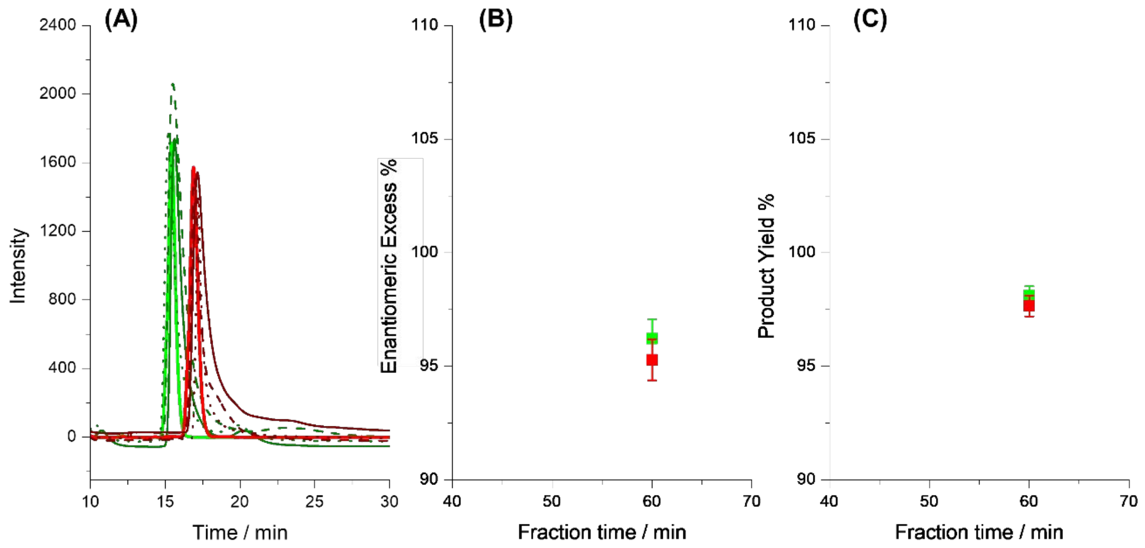

Figure S11. (A) Chromatograms of the fractions collected after 60 minutes from the δ^+ extremity of oligo-(*R*)- and oligo-(*S*)-BT_2T_4/Ppy tubes (green and red, respectively) under an applied electric field of 1.4 V/cm. The solid green and red lines correspond to pristine (*R*)- and (*S*)-PE, respectively. (B) Apparent product yield and (C) enantiomeric excess for six independent experiments, each conducted for one hour: three using Ppy tubes modified with oligo-(*R*)-BT_2T_4 (green) and three with oligo-(*S*)-BT_2T_4/Ppy tubes (red). Data are presented as the mean values of three repetitions for each BT_2T_4/Ppy enantiomers.

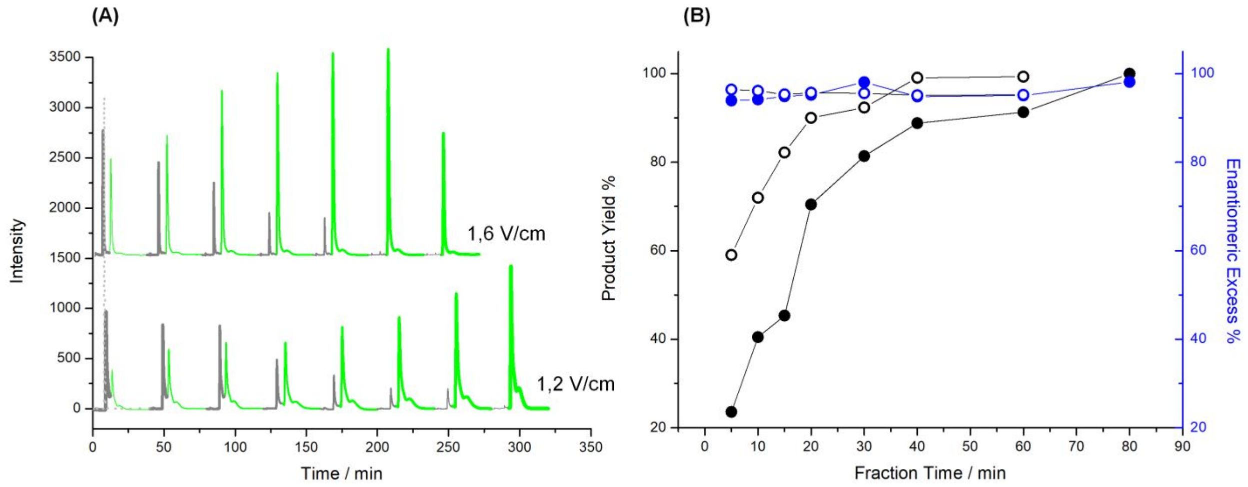

Figure S12. (A) Chromatograms of the fractions collected at different reaction times (5, 10, 15, 20, 30, 40, 60 minutes, as well as 80 minutes for the 1.2 V/cm case) from the δ^+ extremity of oligo-(*R*)-BT_2T_4/Ppy tubes. The green colour stands for (*R*)-PE, while the grey peaks are related to the AP. For comparison, the grey dotted line represents the AP concentration prior to injection into the hybrid tube. (B) Enantiomeric excess (in blue) and apparent product yield (in black) as a function of electrolysis time. Solid dots correspond to the 1.2 V/cm electric field, while empty dots represent the 1.6 V/cm case.

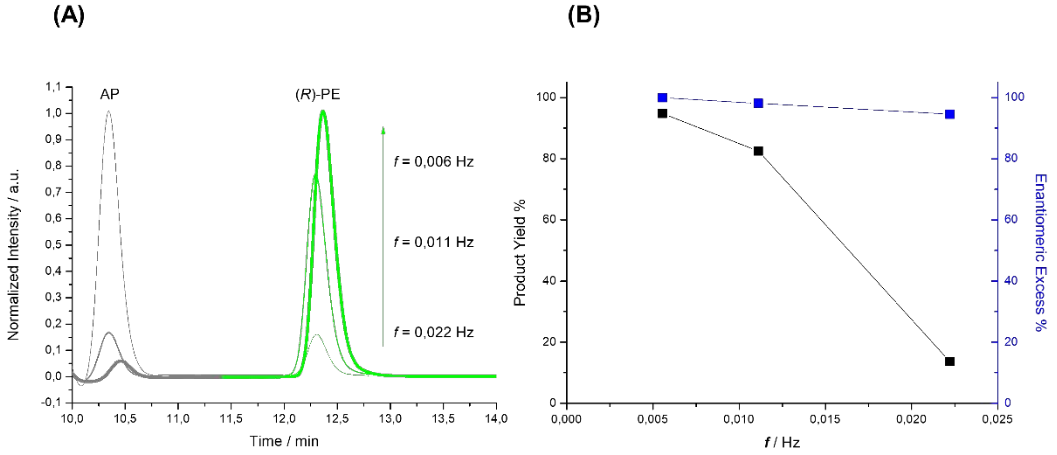

Figure S13. Results related to the alternating current asymmetric electrosynthesis at a constant electric field of 2.8 V/cm as a function of the applied frequency. (A) Chromatograms of the fractions collected after 30 minutes at different applied frequencies (0.006 Hz, 0.011 Hz and 0.022 Hz) from the δ^+ extremity of oligo-(*R*)-BT_2T_4/Ppy tubes. The green color stands for (*R*)-PE, while the grey peaks are related to AP. (B) Enantiomeric excess (in blue) and conversion yield (in black) as a function of the applied frequency.

UV-Vis Spectra for Yield Calculation Validation

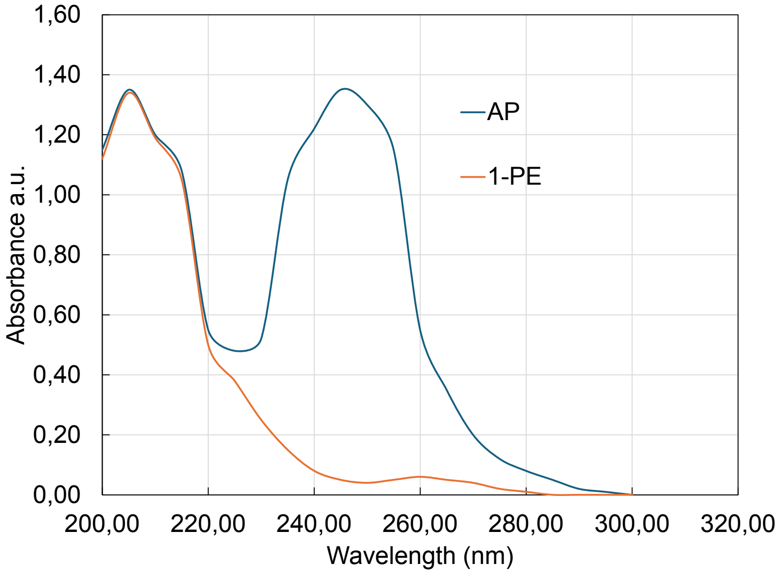

Figure S14. Overlaid UV-Vis absorption spectra of acetophenone and 1-phenylethanol in the HPLC mobile phase (hexane/2-propanol 99:1). The spectra demonstrate nearly identical molar absorptivity at the detection wavelength of 210 nm, validating the use of relative peak area normalization for yield calculation.

Experimental Section

Synthesis of the enantioselective soft tubes

The chiral tubular devices were designed by performing first the potentiodynamic electropolymerization of the enantiopure BT_2T_4 (0.75 mM in ACN, 36 cycles, v = 200 mV s^{-1}) on the surface of a gold wire (Au, ø = 0.3 mm), in a classic three-electrode electrochemical cell, using a Pt wire and an Ag/AgCl as counter and reference electrodes, respectively. Afterwards, the galvanostatic electropolymerization of 0.2 M pyrrole monomer (400 mA for 3600 s) in aqueous solution + 0.25 M sodium dodecylbenzensulfonate (DBS) was carried out. The so obtained chiral tubes were then mechanically removed from the template wire after dipping it in acetone for 15 min.

Wireless enantioselective electrosynthesis

AP and PE. For the wireless enantioselective electrosynthesis experiments, 1-cm long enantiopure soft tubes based on polypyrrole modified at the inner part with oligo-(*S*)- or oligo-(*R*)-BT_2T_4 were fixed in the middle of a classic bipolar cell on an inert support. Two graphite feeder electrodes were positioned at the extremities of the cell (5 cm apart). The electrolyte solution was a pH 4 buffer + 0.2 M $LiClO_4$ as supporting electrolyte to provide enough ions for the electromechanical charge compensation mechanism of polypyrrole. 10 µL of the prochiral precursor (AP) were approached manually as such to the δ^- side of the tube functionalized with oligo-(*S*)- or oligo-(*R*)-BT_2T_4 through a micropipette. The measurements were carried out at three different electric fields, particularly 1.2 V cm^{-1}, 1.4 V cm^{-1} and 1.6 V cm^{-1}. However, to increase the global time of the experiment, we changed the polarity of the feeder electrodes every 15 minutes for 4 consecutive times. All the released fractions were collected manually from the δ^+ end using a microsyringe at different reaction times (5, 10, 15, 20, 30, 40, 60 and 80 minutes). Afterwards, the products were extracted using heptane (1.5 mL) and analyzed by HPLC. Reproducibility tests were performed by repeating the same

measurements with oligo-(S)- and oligo-(R)-modified tubes under a constant electric field of 1.4 V/cm, with manual polarity inversion every 15 minutes. The reaction yield and enantiomeric excess were monitored to assess the consistency of the system. Experiments were monitored by using a CCD camera (CANON EOS R7, Objective Canon Macro Lens 100 mm 1:2.8). Images were processed with ImageJ software.

Lans-S and Lans. For the wireless enantioselective electrosynthesis experiments, 1-cm long enantiopure soft tubes based on Ppy modified at the inner part with oligo-(S)- or oligo-(R)-BT_2T_4 were fixed in the middle of a classic bipolar cell on an inert support. Two graphite feeder electrodes were positioned at the extremities of the cell (5 cm apart). The electrolyte solution was a pH 4 buffer + 0.2 M $LiClO_4$ as supporting electrolyte to provide a sufficient concentration of ions for the electromechanical charge compensation mechanism of Ppy. The Lans-S (2 mM) was dissolved in an achiral ionic liquid, 1-butyl-3-methylimidazolium bis(trifluoromethylsulfonyl)imide (CAS: 174899-83-3; Aldrich 98%), used to avoid the spontaneous diffusion of the probe in the aqueous medium. 10 μL of the reduced form of lansoprazole were manually introduced into the tube with a micropipette. The experiments were carried out under an applied electric field of 1.6 V/cm, with manual polarity inversion of the feeder electrodes every 15 minutes for a total reaction time of 90 minutes. In the case of the (R)-BT_2T_4-modified tube, two fractions were collected manually from the δ^- end using a microsyringe at 45 and 90 minutes. The collected fractions were subsequently extracted using acetonitrile (1.5 mL) and analyzed by HPLC to determine reaction yield and enantiomeric excess.

To ensure the highest precision and reproducibility, all quantitative AC experiments reported herein were conducted using a programmable power supply controlled by a function generator. While the fundamental principle of AC-enhanced conversion was initially verified via manual switching, the automated setup was used for all systematic studies to guarantee the accuracy of the frequency-dependent data.

Reductive Amination of Acetylferrocene. Acetylferrocene (95%, 1271-55-2) and dimethylamine (2.0 M solution in THF, 124-40-3) were purchased from Aldrich and used without further purification. The achiral ionic liquid was also sourced from Aldrich.

A concentrated, self-contained reaction medium was prepared for injection. In a vial, acetylferrocene (11.4 mg, 0.05 mmol) was dissolved in 5 μL of the ionic liquid. To this mixture, dimethylamine (2.0 M solution in THF, 25 μL, 0.05 mmol, 1.0 equiv) was added. The use of the ionic liquid was critical to ensure the co-localization of the reactants and prevent their premature diffusion into the aqueous bulk electrolyte.

For the wireless electrosynthesis, a 1-cm long oligo-(R)-BT_2T_4/Ppy hybrid tube was fixed in the middle of a classic bipolar cell on an inert support. Two graphite feeder electrodes were positioned at the extremities of the cell (5 cm apart). The electrolyte solution consisted of a pH 4 buffer containing 0.2 M $LiClO_4$. A 10 μL droplet of the prepared acetylferrocene/dimethylamine/ionic liquid mixture was manually introduced into the cathodic openting of the tube using a micropipette.

The reaction was carried out under an applied electric field of 1.4 V/cm. To maximize conversion, an alternating current (AC) protocol was employed using a programmable power supply, with the polarity of the feeder electrodes inverted every 15 minutes for a total reaction time of 90 minutes. At the end of the experiment, the liquid expelled from the anodic opening was collected manually with a microsyringe for subsequent workup and analysis.

Chiral HPLC analyses

AP and PE. Chiral HPLC analyses were carried out with a HPLC equipment (Agilent 1260 Infinity II) coupled with a Daicel CHIRALPAK IG-3 column in isocratic reverse phase conditions. The HPLC analyses of the collected fractions at both different reaction times and applied electric fields were carried out by injecting 10 μL of each solution in the chiral column by using hexane/2-propanol (Hex/IPA) 99:1 as the mobile phase and a 0.5 mL/min flow. The photodiode array (PDA) detector was operating at a wavelength of 210 nm. Moreover, acetophenone (*i.e.* the prochiral precursor), the racemate as well as the two antipodes of 1-phenylethanol were also tested separately as such, under the same conditions in order to compare the enantioseparation results with the starting material and to correctly assign the chromatographic peaks to the corresponding enantiomers. The fractions collected after 30 minutes from the AC experiments, performed with 0.5-cm long tubes with varying periods, were extracted with heptane and analyzed using a Jasco high-performance liquid chromatography (HPLC) system (LC-4000) equipped with photodiode array (PDA) and circular dichroism (CD) detectors set at 210 nm and 260 nm, respectively. The HPLC analysis was conducted using a Chiralpak IB N-5 chiral column (250 × 4.6 mm, 5 μm) and an n-heptane/2-propanol mixture (92/8, v/v) as the mobile phase, with a flow rate of 0.5 mL min^{-1}.

Lans-S and Lans. Chiral HPLC analyses were carried out with HPLC equipment (Agilent 1260 Infinity II) and a Daicel CHIRALPAK® IG-3 column in isocratic reverse phase conditions. The HPLC analyses of lansoprazole racemate, lansoprazole sulfide, and sulfone were performed by injecting 30 μL of each solution in the chiral column, with ACN/H_2O 55:45 as eluent and a 0.5 ml/min flow, T= 40°C. The photodiode array (PDA) detector was operating at a wavelength of 280 nm. To compare the results, the three compounds were tested separately and simultaneously under the same conditions.

Reductive Amination of Acetylferrocene. The collected fractions were transferred to a separation funnel, basified with 1 M NaOH (aq.), and extracted with diethyl ether (3 x 1.5 mL). The combined organic layers were dried over anhydrous Na_2SO_4, filtered, and the solvent was removed under reduced pressure to yield the crude product as a dark red oil.

Chiral High-Performance Liquid Chromatography (HPLC) analyses were performed on an Agilent 1260 Infinity II system equipped with a photodiode array (PDA) detector. The enantiomers of N,N-dimethyl-1-ferrocenylethylamine were separated using a Daicel CHIRALPAK® IG-3 column under isocratic conditions. The mobile phase consisted of a mixture of hexane/ethanol/dichloromethane (99:1:0.1, v/v), delivered at a flow rate of 1.0 mL/min. The column temperature was maintained at 25 °C. Detection was performed at 420 nm (characteristic of the ferrocene *d-d* electronic transition). Under these conditions, the retention times were 5 min for the (*R*)-enantiomer and 7 min for the (*S*)-enantiomer. The enantiomeric excess (ee) was calculated from the integrated peak areas using the standard formula.

Electrochemical Quartz Crystal Microbalance (EQCM) Measurements

EQCM experiments were performed using a quartz crystal microbalance (EQCM 5710, Institute of Physical Chemistry of the Polish Academy of Sciences, Warsaw, Poland) connected to a potentiostat. AT-cut quartz crystals with a fundamental frequency of 10 MHz, coated with gold electrodes, were used as the working electrodes. The chiral interface was prepared by potentiodynamic electropolymerization of the (*R*)- or (*S*)-BT_2T_4 monomer directly onto the gold surface, following the same procedure used for the reactor fabrication. The modified crystal was mounted in a flow-cell setup. A stable baseline frequency was first established in the electrolyte solution (pH 4 buffer + 0.2

M $LiClO_4$). Subsequently, a solution containing the chiral probe (2 mM (*R*)- or (*S*)-N,N-dimethyl-1-ferrocenylethylamine) was injected into the cell, and the change in resonant frequency (Δf) was monitored over time to quantify the mass uptake corresponding to the preferential adsorption of one enantiomer.

Solid-State NMR (ssNMR) Spectroscopy

Solid-state ^{13}C NMR spectra were recorded on a solid-state NMR spectrometer. Samples for interaction studies were prepared by incubating the Ppy/oligo-BT_2T_4 composite material with a solution of the chiral probe ((*R*)- or (*S*)-Fc) for 12 hours. The material was then thoroughly washed with the solvent to remove any unbound probe, dried under vacuum, and packed into 3.2 mm or 4.0 mm zirconia rotors with magnesium oxide (MgO) used as a filler. All spectra were acquired at a nominal temperature of 295 K or 298 K using a magic angle spinning (MAS) speed of 15 kHz. ^{13}C Cross-Polarization (cpmas) spectra were acquired using a contact time of 5.0 ms and a recycle delay of 5 s. ^{13}C direct polarization spectra were acquired using high-power proton decoupling (hpdec) with a recycle delay of 5 s. Chemical shifts were referenced externally to a standard sample of adamantane.

Magnetic Circular Dichroism (MCD) Spectroscopy

MCD spectra were recorded on a Jasco [J-815] spectropolarimeter equipped with a 1.5 T permanent magnet. For the analysis of the diastereomeric interaction, a thin film of the (*S*)-oligo-BT_2T_4 was first deposited via potentiodynamic electropolymerization onto a transparent quartz slide. The slide was then placed in a custom-made cuvette inside the sample compartment of the spectrometer. A baseline spectrum was recorded with the cuvette filled with the electrolyte solution (pH 4 buffer + 0.2 M $LiClO_4$). Subsequently, a solution of the chiral probe ((*R*)- or (*S*)-Fc) was added to the cuvette to a final concentration of 1 mM. MCD spectra were recorded from 600 nm to 350 nm at a scan speed of 100 nm/min, with an accumulation of 5 scans. The experiment was repeated for both enantiomers of the ferrocene probe to compare the spectral signatures of the "matching" and "mismatching" interactions.

Intra-tubular Flow Quantification by Micro-Particle Tracking Velocimetry (μ-PTV)

The fluid dynamics inside the 1 cm oligo-BT_2T_4/Ppy hybrid tube were quantified using Micro-Particle Tracking Velocimetry (μ-PTV). The tube was fixed on an inert support within a bipolar cell containing a pH 4 buffer with 0.2 M $LiClO_4$ as the electrolyte. Two graphite feeder electrodes were positioned 5 cm apart to apply a constant electric field. To visualize the flow, the electrolyte was seeded with fluorescent tracer particles. A microscope equipped with a CCD camera (CANON EOS R7) was focused on the center of the tube to record the movement of these particles.

The experiments were conducted under three different applied electric fields: 1.2, 1.4, and 1.6 V/cm. Video frames were processed using ImageJ software to track the particle velocity at different radial positions (r) from the tube's center (r=0) to the wall (r=100 μm). From the measured parabolic velocity profiles, the volumetric flow rate (Q) was calculated by integrating the velocity profile across the tube's cross-section (approximated as $Q=0.5\cdot\pi\cdot R^2\cdot v_{max}$). The effective pressure gradient ($\Delta P/L$) generated by the electromechanical actuation was then rigorously calculated from the flow data using the Hagen-Poiseuille equation: $\Delta P/L=(8\cdot\mu\cdot Q)/(\pi\cdot R^4)$, where μ is the fluid viscosity.

Synchronized Electrochemical and Flow Data Acquisition

To establish a direct causal link between ion flux and fluid pumping, a synchronized experiment combining chronoamperometry and time-resolved μ-PTV was performed. The reactor was set up in

the bipolar cell as described above, under a constant applied electric field of 1.4 V/cm. The current transient (I(t)), which is a direct measure of the ion flux into the polymer actuator, was recorded using a potentiostat. Simultaneously, the resulting fluid flow rate (Q(t)) was measured using time-resolved μ-PTV.

The total electrical charge passed (q(t)) was calculated by integrating the current over time ($q=\int I \cdot dt$). Similarly, the cumulative volume of fluid displaced (V(t)) was calculated by integrating the instantaneous flow rate over time ($V=\int Q \cdot dt$). The resulting data were used to generate a parametric plot of cumulative displaced volume versus cumulative charge, from which the device's pumping efficiency (in nL/μC) was determined from the slope of the linear, steady-state regime.

Thermodynamic Analysis of Enantioselectivity

To provide a quantitative understanding of the energy barriers governing the enantioselective reaction, temperature-dependence studies were conducted. The enantioselective reduction of AP was performed at different temperatures, and the resulting enantiomeric excess (ee) was used to determine the differential activation parameters ($\Delta\Delta H^{‡}$ and $\Delta\Delta S^{‡}$) through an Eyring analysis.

All temperature-dependent experiments were performed using the AC protocol in the same bipolar cell configuration described above. A 1-cm long oligo-(*S*)-BT_2T_4/Ppy hybrid tube was fixed between two graphite feeder electrodes (5 cm apart) in a cell containing a pH 4 buffer with 0.2 M $LiClO_4$. The entire bipolar cell was submerged in a thermostated water bath equipped with a cryostat to maintain a constant temperature. The temperature of the electrolyte solution was monitored with a calibrated digital thermometer and was stable to within ±0.2 °C of the set point for the duration of each experiment. The enantioselective reduction of AP was carried out at five different temperatures: 0 °C, 10 °C, 20 °C, 30 °C, and 40 °C. For each experiment, the system was allowed to thermally equilibrate at the target temperature for 20 minutes. A 10 μL droplet of AP was then introduced at the cathodic end of the hybrid tube. An electric field of 1.4 V/cm was applied using a programmable power supply, with the polarity inverted every 15 minutes for a total reaction time of 60 minutes to ensure high conversion. At the end of the experiment, the liquid expelled from the tube was collected for chiral HPLC analyses (detailed in the dedicated experimental section). The procedure was repeated for each temperature point. The enantiomeric excess was calculated from the integrated peak areas of the (*R*)- and (*S*)-enantiomers using the formula provided below in the Supporting Information.

Macroscope images

Macroscope images were recorded with a digital Leica Z16 APO model.

Video S1. Alternating current-driven liquid flow (10 times accelerated)

Video S2. Alternating current-driven liquid flow at different applied frequencies (10 times accelerated)

Quantification of Yield and Enantiomeric Excess

Conversion yields and enantiomeric excess (*ee*) were determined from the integrated peak areas in the HPLC chromatograms. The following formulas were used:

$$Conversion\,Yeld\,(\%) = \frac{Peak\,area_{product}}{Peak\,area_{product} + Peak\,area_{reactant}} x100$$

$$ee\ (\%) = \frac{|Peak\ area_{R-enantiomer} - Peak\ area_{S-enantiomer}|}{Peak\ area\ _{R-enantiomer} + Peak\ area_{S-enantiomer}} x100$$

This relative area normalization method is justified for monitoring the kinetic profile of the reaction, as the reactant (AP) and product (PE) have nearly identical chromophores and thus very similar molar extinction coefficients at the detection wavelength (210 nm), as confirmed by UV-Vis spectroscopy (see Figure S14). All synthesized products were characterized by ^{1}H NMR, ^{13}C NMR, and HRMS, and the data, which matched literature values, are provided in Table S1.

Table S1. Characterization Data for Synthesized Products and Comparison with Literature

Compound	Technique	Experimental Data	Literature Data	Source
**(*R*)-1-Phenylethanol**	^{1}H NMR ($CDCl_3$, 400 MHz)	δ 7.38–7.25 (m, 5H, Ar-H), 4.90 (q, J=6.5 Hz, 1H, CH), 1.95 (br s, 1H, OH), 1.49 (d, J=6.5 Hz, 3H, CH_3).	δ 7.37–7.26 (m, 5H), 4.89 (q, 1H), 2.1 (s, 1H), 1.49 (d, 3H).	[1]
	^{13}C NMR ($CDCl_3$, 100 MHz)	δ 145.7, 128.6, 127.5, 125.4, 70.3, 25.1.	δ 145.8, 128.5, 127.4, 125.4, 70.4, 25.2.	[2]
	HRMS (ESI+)	m/z $[M+Na]^+$ calcd for $C_8H_{10}ONa$: 145.0624; found: 145.0621.	m/z $[M]^+$ calcd for $C_8H_{10}O$: 122.0732.	[3]
**d-Lansoprazole**	^{1}H NMR (DMSO-d_6, 400 MHz)	δ 13.7 (br s, 1H, NH), 8.26 (d, J=5.2 Hz, 1H), 7.69 (m, 1H), 7.58 (m, 1H), 7.32 (m, 2H), 7.11 (d, J=5.2 Hz, 1H), 4.88 (q, J=8.8 Hz, 2H), 4.75 (ABq, 2H), 2.16 (s, 3H).	δ 8.25 (d, 1H), 7.6-7.8 (m, 2H), 7.3-7.4 (m, 2H), 7.1 (d, 1H), 4.8-5.0 (q, 2H), 4.6-4.8 (m, 2H), 2.15 (s, 3H).	[4]
	HRMS (ESI+)	m/z $[M+H]^+$ calcd for	m/z $[M+H]^+$ calcd for	[5]

		$C_{16}H_{15}F_3N_3O_2S$: 370.0837; found: 370.0835.	$C_{16}H_{15}F_3N_3O_2S$: 370.0837.	
(*R*)-N,N-dimethyl-1-ferrocenylethylamine	^{1}H NMR ($CDCl_3$, 600 MHz)	δ 4.19–4.08 (m, 9H, Cp-H), 3.81 (q, J=6.8 Hz, 1H, CH-N), 2.17 (s, 6H, $N(CH_3)_2$), 1.53 (d, J=6.8 Hz, 3H, CH_3).	δ 3.97–4.19 (m, 9H), 3.59 (q, J=6.9 Hz, 1H), 2.08 (s, 6H), 1.44 (d, J=6.9 Hz, 3H).	[6]
	^{13}C NMR ($CDCl_3$, 151 MHz)	δ 93.8 (C-ipso), 68.3 (Cp-unsub.), 67.8, 67.5, 67.1, 66.5 (Cp-sub.), 58.4 (CH-N), 41.1 ($N(CH_3)_2$), 14.9 (CH_3).	δ 93.9, 68.4, 67.9, 67.6, 67.2, 66.7, 58.5, 41.2, 15.0.	[7]
	HRMS (ESI+)	m/z $[M+H]^+$ calcd for $C_{14}H_{20}FeN$: 258.0940; found: 258.0938.	m/z $[M]^+$ calcd for $C_{14}H_{19}FeN$: 257.0867.	/

1. https://spectrabase.com/spectrum/Gl2XXBDCxi3
2. *Nanoscale Adv.*, 2021,3, 3306-3315
3. https://www.chemicalbook.com/SpectrumEN_1517-69-7_13cnmr.htm
4. https://nmr.oxinst.com/assets/uploads/MagRes/App%20Notes/X-Pulse_Application_Note_29_Characterization_of_Lansoprazole_by_Benchtop_NMR_Spectroscopy.pdf
5. *American Journal of Analytical Chemistry*, 6, 145-155.
6. *Synthesis (Stuttg).*, 2023;55, 2390-2396.
7. *Chem. Soc. Rev.*, 2004, 33, 313-328.

Table S2. Post-Mortem Analysis of Reactor Stability and Degradation.

Quantitative analysis of the reactor's performance and electrochemical properties over three consecutive one-hour reaction cycles. The significant drop in reaction yield after the second cycle correlates directly with a sharp increase in the charge-transfer resistance (R_{ct}) of the Ppy actuator (measured by EIS) and a corresponding decrease in its redox peak current (measured by CV). In contrast, the peak current of the inner oligo-BT_2T_4 remains stable, confirming that the primary failure mode is the irreversible over-oxidation of the Ppy actuator, while the chiral catalyst layer remains unchanged.

Parameter	Technique	Pristine Device (Cycle 0)	After Cycle 1	After Cycle 2	After Cycle 3 (Degraded)
Reaction Yield (%)	HPLC	99	97	95	64

Charge-Transfer Resistance (R_{ct}) - Ppy Actuator (Ω)	EIS	550	680	1150	4800
Peak Current (CV) - Ppy Actuator (μA)	CV	150	142	115	38
Peak Current (CV) - Oligo-BT_2T_4 (μA)	CV	80	79	78	76

Theoretical Framework for Electro-Chemo-Mechanical Pumping

The actuation of conducting polymers like Ppy is a well-documented phenomenon driven by the reversible exchange of ions and solvent molecules with an electrolyte, which occurs during electrochemical oxidation and reduction. These mass transfers induce volumetric changes in the polymer matrix, providing the basis for their use as artificial muscles and actuators.

In our Ppy/DBS system, the large dodecylbenzenesulfonate (DBS^-) anions are immobilized within the polymer matrix during synthesis. Consequently, charge neutrality during redox cycling is maintained by the flux of smaller, solvated cations (Li^+) from the aqueous $LiClO_4$ electrolyte. The reduction of the Ppy backbone at the cathodic pole (δ^-) necessitates an influx of Li^+ ions, causing local swelling. Conversely, oxidation at the anodic pole (δ^+) causes an efflux of Li^+ ions, leading to local shrinking. To a first approximation, the resulting volumetric strain (ε_v) is linearly proportional to the density of injected charge (ρ_q). This fundamental relationship forms the core of our electro-chemo-mechanical model:

$$\varepsilon_v = \alpha \rho_q = \alpha \frac{\Delta Q}{V_{poly}}$$

where α is the strain-to-charge coefficient (in units of m^3/C), which represents the volume change per unit of charge injected, ΔQ is the total charge transferred (in C), and V_{poly} is the volume of the polymer actuator (in m^3). This equation provides the essential link between the electrochemical stimulus (ΔQ) and the mechanical response (ε_v).

To translate this volumetric strain into a net fluid flow, a more sophisticated model is required that accounts for the geometry of the tube and the anisotropic nature of the deformation. A simple isotropic swelling would cause the tube to shorten and thicken, which is not an efficient mechanism for pumping. The observed unidirectional flow relies on an anisotropic deformation, specifically a change in the tube's inner radius that is more pronounced than any change in its length. This anisotropy, arising from the polymer chain alignment and the geometric constraints of the tube, is what generates the axial pressure gradient responsible for pumping. We propose a multiphysics simulation framework, for instance within the COMSOL Multiphysics environment, to model this complex interplay.

A 2D axisymmetric model would be sufficient and would couple four distinct physics interfaces:

1. **Electrostatics:** This module solves for the electric potential (ϕ) in the electrolyte surrounding the BPE. It establishes the potential difference (ΔV) induced across the conductive polymer

tube, which in turn defines the local potential at the polymer-electrolyte interface along the tube's length, z.

2. **Transport of Diluted Species (Nernst-Planck Equations):** This interface models the flux of Li^+ ions (N_{Li+}) into and out of the polymer matrix. The flux is driven by both electromigration (due to the electric field gradient) and diffusion (due to the concentration gradient), allowing for the calculation of the local charge density, ρ_q (z, t), as a function of position and time.

3. **Solid Mechanics:** This module treats the Ppy tube as an elastic solid. The crucial step is the multiphysics coupling, where the charge density calculated in the transport module is used to define an anisotropic, ion-induced strain tensor, ε_{ion}.

   Based on the requirement for anisotropic deformation, this tensor can be defined as:

$$\varepsilon_{ion} = \begin{pmatrix} \varepsilon_{rr} & 0 & 0 \\ 0 & \varepsilon_{\theta\theta} & 0 \\ 0 & 0 & \varepsilon_{zz} \end{pmatrix} = \begin{pmatrix} \frac{1}{3}\alpha_{rad}\rho_q(z,t) & 0 & 0 \\ 0 & \frac{1}{3}\alpha_{rad}\rho_q(z,t) & 0 \\ 0 & 0 & \frac{1}{3}\alpha_{ax}\rho_q(z,t) \end{pmatrix}$$

   Here, the radial strain coefficient (α_{rad}) is set to be significantly larger than the axial strain coefficient (α_{ax}) to reflect the preferential radial expansion and contraction that drives the pumping.

4. **Laminar Flow (Navier-Stokes Equations):** This interface solves for the fluid velocity (u) and pressure (p) inside the tube. A "Moving Mesh" feature is employed, where the boundary of the fluid domain (the inner wall of the tube) is dynamically updated by the deformed geometry calculated in the Solid Mechanics module. The asymmetric radial deformation, swelling at the cathodic end and shrinking at the anodic end, creates a net pressure gradient that drives the fluid from the cathode to the anode, consistent with our experimental observations and the Bernoulli principle.

To demonstrate the feasibility of this simulation, we have compiled a table of key physical parameters required for its implementation, based on values reported in the literature for similar systems.

Table S3. Key Parameters for Multiphysics Simulation of Electromechanical Pumping

Parameter	Symbol	Value	Unit	Source/Reference
Young's Modulus (Ppy)	E_{Ppy}	0.5 - 2.0	GPa	4
Poisson's Ratio (Ppy)	ν_{Ppy}	0.35	-	Assumed (typical for polymers)
Strain-to-Charge Ratio	α	$\sim 1\times10^{-10}$	m^3/C	2
Diffusion Coeff. (Li^+ in Ppy)	D_{Li+}	10^{-12}–10^{-10}	m^2/s	5

Electrolyte Conductivity	σ_{elec}	~2	S/m	Experimental (0.2 M $LiClO_4$)
Fluid Density (Electrolyte)	ρ_{fluid}	~1000	kg/m^3	Water approximation
Fluid Viscosity (Electrolyte)	μ_{fluid}	~1×10^{-3}	Pa·s	Water approximation

References

1. Conducting polymer actuators as engineering materials, https://people.ece.ubc.ca/mm/papers/Madden_SPIE_2002.pdf
2. Polypyrrole actuators: Modelling and performance, https://www.researchgate.net/publication/252688503_Polypyrrole_actuators_Modelling_and_performance
3. Multi-physical modeling for electro-transport and deformation of ionic polymer metal composites, https://www.spiedigitallibrary.org/conference-proceedings-of-spie/8340/83400Q/Multi-physical-modeling-for-electro-transport-and-deformation-of-ionic/10.1117/12.913020.pdf
4. Development and characterization of conducting polymer actuators, https://www.researchgate.net/publication/279819776_Development_and_characterization_of_conducting_polymer_actuators
5. The Relation of Conducting Polymer Actuator Material Properties to Performance, https://people.ece.ubc.ca/mm/papers/PMadden_IEEE_2004.pdf

Table S4. Intra-tubular Flow Quantification (μ-PTV) measuring the fluid velocity inside the tube at different applied electric fields and calculating the effective pumping pressure.

**A: Model Parameters**			
Inner Radius (R)	100	μm	
Fluid Viscosity (μ)	0.01	Pa·s	
**B: Velocity Profiles (μ-PTV Measurement)**			
**Radial Position r (μm)**	**Velocity v (μm/s) @ 1.2 V/cm**	**Velocity v (μm/s) @ 1.4 V/cm**	**Velocity v (μm/s) @ 1.6 V/cm**
0	251.3	402.1	598.5
10	248.1	397.5	593.1
20	242.0	385.0	577.3
30	225.7	361.1	541.2

40	211.1	335.8	505.9
50	189.2	302.7	451.6
60	161.5	255.3	380.8
70	122.8	198.1	296.4
80	90.1	142.9	211.7
90	48.9	78.2	118.3
100	0.0	0.0	0.0
**C: Calculated Hydrodynamic Properties**			
**Applied Field (V/cm)**	**Max Velocity v_{max} (µm/s)**	**Volumetric Flow Rate Q (nL/s)**	**Effective Pressure Gradient (Pa/m)**
1.2	251.3	4.35	100.5
1.4	402.1	6.32	160.8
1.6	598.5	9.40	239.4

A (Model Parameters): These are the known physical constants of the system: the inner radius of the Ppy tube (~100 µm) and the viscosity of the aqueous electrolyte, which is similar to water.

B (Velocity Profiles): This table shows the data from the µ-PTV experiments. A microscope was focused on the center of the tube and fluorescent particles were tracked to measure their speed at different radial positions (r), from the center (r=0) to the wall (r=100 µm).

The obtained **parabolic velocity profile** is the classic signature of pressure-driven laminar flow (Poiseuille flow) in a tube. The velocity is maximum at the center and zero at the wall due to friction.

Crucially as the applied electric field increases from 1.2 to 1.6 V/cm, the maximum velocity (v_{max}) increases significantly. This directly visualizes the effect of the electrical stimulus on the fluid motion.

C (Calculated Hydrodynamic Properties): Summary table where the raw velocity data is converted into some key physical quantities:

Volumetric Flow Rate (Q): This is calculated by integrating the velocity profile across the tube's cross-section. For a parabolic profile, this simplifies to the formula $Q=0.5\cdot\pi\cdot R^2\cdot v_{max}$. The results show that the pumping rate increases from ~4 nL/s to over 9 nL/s as the field strength is increased.

Effective Pressure Gradient (ΔP/L): This is the most critical result. While you cannot easily measure this pressure directly, it can be calculated from the flow data using the Hagen-Poiseuille equation: $\Delta P/L=(8\cdot\mu\cdot Q)/(\pi\cdot R^4)$.

Table S5. Synchronized Electrochemical & Flow Data

**Transient Data (at 1.4 V/cm)**				
**Time (s)**	**Current I(t) (μA)**	**Flow Rate Q(t) (nL/s)**	**Cumulative Charge q(t) (μC)**	**Cumulative Volume V(t) (nL)**
0.0	0.0	0.0	0.0	0.0
0.5	122.8	2.5	46.4	0.6
1.0	100.6	4.0	102.2	2.3
1.5	82.4	5.0	148.2	4.5
2.0	67.5	5.6	186.1	7.1
2.5	55.3	5.9	216.8	10.0
3.0	45.3	6.1	242.0	13.0
3.5	37.1	6.2	262.6	16.1
4.0	30.4	6.3	279.5	19.2
4.5	24.9	6.3	293.3	22.4
5.0	20.4	6.3	304.6	25.5
5.5	16.7	6.3	313.9	28.7
6.0	13.7	6.3	321.5	31.8
6.5	11.2	6.3	327.7	35.0
7.0	9.2	6.3	332.8	38.1
7.5	7.5	6.3	337.0	41.3
8.0	6.2	6.3	340.4	44.4
8.5	5.0	6.3	343.2	47.6
9.0	4.1	6.3	345.5	50.7
9.5	3.4	6.3	347.4	53.9
10.0	2.8	6.3	348.9	57.0

Current I(t)) values are related to chronoamperometry experiments. When the voltage is first applied, there is a large initial current as ions rush to the polymer surface. This current then decays exponentially as the polymer charges and ion diffusion becomes the limiting factor. This is a classic Cottrell-like decay.

Flow Rate Q(t) data comes from the time-resolved μ-PTV measurements. The flow does not start instantaneously. There is a slight lag as the polymer needs time to swell and accelerate the fluid. The flow rate then rises and saturates at a steady-state value (here, ~6.3 nL/s)

The Cumulative Charge q(t)) is the integral of the current over time ($q=\int I\cdot dt$). It represents the total amount of charge, and therefore the total number of ions, that has moved into the polymer matrix up to that point in time.

Cumulative Volume V(t)) is the integral of the flow rate over time ($V=\int Q\cdot dt$). It represents the total volume of fluid that has been pumped out of the tube up to that point in time.

The linear relationship proves the mechanism demonstrating a direct, proportional causality: for every unit of charge (representing a fixed number of ions) that enters the polymer, a fixed unit of volume is pumped. The slope of this line gives the pumping efficiency that is approximately 57.0 nL / 348.9 μC = 0.16 nL/μC.

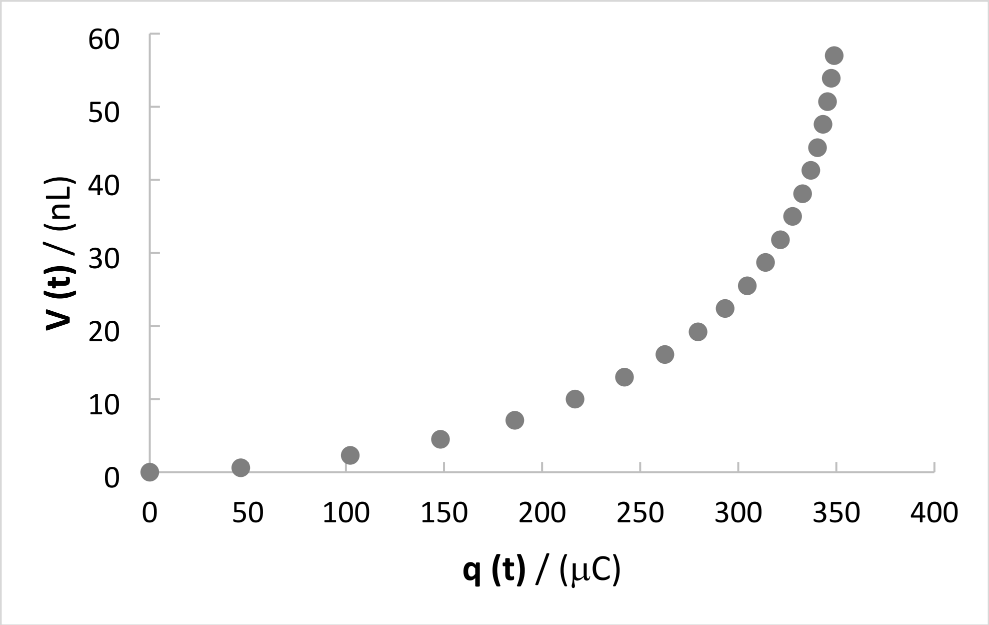

Figure S15. Parametric plot of cumulative displaced volume versus cumulative charge, establishing a direct causal link between ion flux and fluid flow. Data points were derived by integrating synchronized current transients (from chronoamperometry) and volumetric flow rates (from time-resolved μ-PTV).

The apparent exponential shape in Figure S15 at the beginning of the plot is due to the different transient responses of the electrochemical and hydrodynamic systems when the voltage is first applied. The current I(t) is very high in the first couple of seconds (122.8 μA at 0.5s) and then decays rapidly. This means that a large amount of charge q(t) is injected into the polymer very quickly at the beginning. This is the "charging" phase of the polymer actuator.

In contrast, the fluid flow Q(t) starts at zero and takes time to accelerate (Figure S15). This is due to the fluid's inertia and the finite time required for the polymer to physically swell and begin pushing the liquid. The flow rate only reaches its constant, steady-state value of 6.3 nL/s after about 4 seconds.

Because the cumulative charge builds up rapidly while the cumulative volume displacement lags behind, the initial part of the Volume vs. Charge graph has a shallower slope. As the system settles into a steady state (roughly after t = 4 seconds), both the rate of charge injection (current) and the rate of fluid flow become stable and constant. In this steady-state regime, the relationship becomes directly proportional and therefore linear. The crucial takeaway from this experiment is the linear relationship observed in this steady-state region. The slope of this linear portion is what defines the fundamental "pumping efficiency" (in nL/μC), as it represents the direct, causal link between a steady ion flux and the resulting steady fluid flow. The initial curve simply captures the start-up dynamics of the system before it reaches stable operation.

Computational Methods

Density Functional Theory (DFT) calculations were performed to elucidate the mechanism and origin of enantioselectivity for the electrochemical reduction of acetophenone and oxidation of lansoprazole sulfide.

Model Construction: The chiral catalyst, (*R*)-oligo-BT_2T_4, was modeled using a truncated dimer of the monomer unit to create a realistic representation of the helical binding pocket while maintaining computational tractability. To simulate the conditions at the polarized cathodic surface, a net negative charge was applied to the oligomer model. The electrochemical environment of the aqueous buffer (pH 4) was represented using the SMD implicit solvent model.

Level of Theory: Geometry optimizations of all stationary points (reactants, transition states, and products) were carried out using the M06-2X functional, which is well-suited for describing the non-covalent interactions critical for stereodifferentiation. The def2-TZVP basis set was employed for all atoms, providing a triple-zeta quality description necessary for accurately modeling the subtle electronic effects that govern enantioselectivity.

Transition State Verification: Transition state (TS) structures were located and characterized by frequency calculations, confirming the presence of a single imaginary frequency corresponding to the desired reaction coordinate. To ensure that each transition state connected the correct reactant and product complexes, Intrinsic Reaction Coordinate (IRC) analyses were performed.

Thermodynamic and Selectivity Analysis: Gibbs free energies (G) were calculated at 298.15 K from the unscaled vibrational frequencies obtained at the M06-2X/def2-TZVP level of theory. The difference in Gibbs free energy between the two diastereomeric transition states ($\Delta\Delta G^{\ddagger} = G_{\text{TS-disfavored}} - G_{\text{TS-favored}}$) was used to predict the enantiomeric excess (ee). The enthalpic ($\Delta\Delta H^{\ddagger}$) and entropic ($\Delta\Delta S^{\ddagger}$) contributions to the activation barrier were also calculated from the vibrational analysis to allow for direct comparison with the experimental Eyring analysis.

Interaction Analysis: To gain qualitative and quantitative insight into the specific interactions responsible for stereocontrol, the optimized transition state structures were analyzed using the Non-Covalent Interaction (NCI) plot method. This technique allows for the visualization of weak interactions, such as C-H···π bonds and steric repulsion, that stabilize the favored transition state and destabilize the disfavored one.

Table S6. Comparison of Experimental and Computationally Stereochemical Outcomes and Thermodynamic Parameters

**Parameter**	**Substrate**	**Experimental Value**	**DFT Value**
**Enantiomeric Excess (ee)**	Acetophenone	>99%	99.8%
	Lansoprazole Sulfide	>90%	94.5%
**ΔΔG‡ (at 298 K)**	Acetophenone	>13.1 kJ/mol	+15.5 kJ/mol
**ΔΔH‡**	Acetophenone	+19.9 kJ/mol	+21.2 kJ/mol
**ΔΔS‡**	Acetophenone	-28.3 J K^{-1} mol^{-1}	-19.1 J K^{-1} mol^{-1}

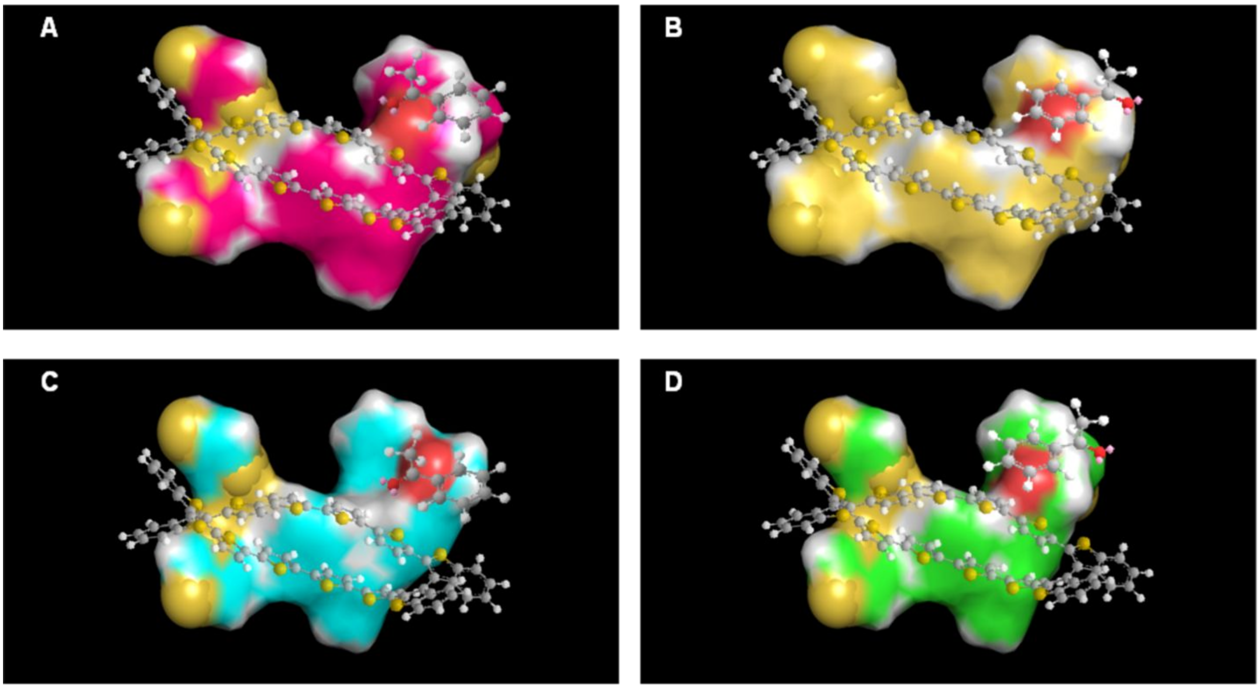

Figure S16. Visualizing the Molecular Origins of Enantioselectivity. This figure provides a direct, atomic-level comparison of the DFT-optimized transition states for the favourable and unfavourable pathways of acetophenone reduction. Two complementary computational analyses are shown: Non-Covalent Interaction (NCI) plots (Panels A and B), which visualize steric and weak attractive forces, and Electrostatic Potential (ESP) maps (Panels C and D), which illustrate the distribution of charge.

- (A) Favourable Transition State, NCI Analysis: The surface is dominated by extensive pink/magenta regions, which represent a network of weak but cumulatively stabilizing non-covalent interactions, including van der Waals forces and the key C-H···π interaction between the substrate's methyl group and the catalyst's chiral pocket. Notably, the strong steric repulsion seen for the unfavourable pathway is absent.
- (B) Unfavourable Transition State, NCI Analysis: This structure is characterized by an intense, localized red region, the signature of a strong, destabilizing steric clash. This

repulsion occurs between the substrate's bulky phenyl group and the backbone of the chiral oligomer, providing a clear visual rationale for the high kinetic barrier of this pathway.

- (C) Favourable Transition State, ESP Analysis: The surface within the catalyst's pocket is predominantly cyan/blue, indicating a region of positive electrostatic potential. This corresponds to the hydrogen atoms of the acetophenone's methyl group. This orientation creates a favourable electrostatic interaction with the electron-rich π-system of the catalyst's thiophene rings, providing further stabilization to this transition state. The red area corresponds to the negative potential on the carbonyl oxygen.
- (D) Unfavourable Transition State, ESP Analysis: In contrast, the catalyst pocket is now filled with a largely green surface, representing the neutral electrostatic potential of the apolar phenyl group. This demonstrates a lack of favourable electrostatic complementarity, confirming that this orientation is not electronically stabilized.

Thermodynamic Analysis via Temperature-Dependence Studies (Eyring Plot)

To dissect the energy barrier ($\Delta\Delta G^‡$) into its enthalpic ($\Delta\Delta H^‡$) and entropic ($\Delta\Delta S^‡$) components, the enantioselective reduction of AP was performed at various temperatures. The enantiomeric excess (ee) was measured at each temperature by chiral HPLC.

Table S7. ee values measured at five different temperatures for the synthesis of (*R*)-PE using the (*R*)-oligo-BT_2T_4 reactor.

Temperature (°C)	Temperature (K)	1/T (K^{-1})	Measured ee (%)	ln((1+ee)/(1−ee))
0	273.15	0.003661	99.55	5.40
10	283.15	0.003532	99.21	4.83
20	293.15	0.003411	98.66	4.29
30	303.15	0.003299	97.80	3.80
40	313.15	0.003193	96.51	3.32

According to the Eyring equation, the plot of ln((1+ee)/(1−ee)) versus 1/T yield a straight line.

$$ln\frac{1+ee}{1-ee} = -\frac{\Delta\Delta H^‡}{R}\frac{1}{T} + \frac{\Delta\Delta S^‡}{R}$$

The linear Eyring plot has a high correlation coefficient (R^2>0.99) with a slope ($-\Delta\Delta H^‡/R$) of approximately −2400 K and a y-intercept ($\Delta\Delta S^‡/R$) of approximately −3.4.

$\Delta\Delta H^‡ = -(\text{slope}\times R) = -(-2400\ K \times 8.314\ J\ K^{-1}\ mol^{-1}) \approx +19.9\ kJ/mol$

$\Delta\Delta S^‡ = (\text{intercept}\times R) = (-3.4\times 8.314\ J\ K^{-1}\ mol^{-1}) \approx -28.3\ J\ K^{-1}mol^{-1}$

A large positive $\Delta\Delta H^‡$ and a negative $\Delta\Delta S^‡$ provide powerful confirmation of the proposed mechanism. This indicates that the selectivity is primarily entropically driven. The negative $\Delta\Delta S^‡$ suggests that the favored transition state is significantly more ordered and conformationally restricted than the disfavored one.

Kinetic Isotope Effect (KIE) Studies

Acetophenone-d_3 was purchased from Merck (CAS 17537-31-4) and used as such without further purification steps. To determine the KIE, a competitive experiment was performed. A stock solution containing an equimolar (1:1) mixture of acetophenone (AP) and acetophenone-d_3 (AP-d_3) was prepared. A 10 µL droplet of this mixture was subjected to the standard AC electrosynthesis protocol using an oligo-(*R*)-BT_2T_4/Ppy tube with an electric field of 1.4 V/cm for 30 minutes to ensure low conversion (<20%). The reaction was stopped, and the collected liquid was extracted with heptane. The relative amounts of remaining starting materials (AP and AP-d_3) and the generated products (1-phenylethanol, PE, and 1-phenylethanol-d_3, PE-d_3) were quantified by GC-MS (Agilent Technologies) to determine the KIE value using the following equation:

$$k_H/k_D = \ln(1 - f_H) / \ln(1 - f_D)$$

where f_H and f_D are the fractional conversions of the non-deuterated and deuterated starting materials, respectively. The enantiomeric excess (ee) of the 1-phenylethanol product was determined by chiral HPLC analysis of a separate, non-competitive reaction run to high conversion (>95%) to ensure accurate ee measurement. The results are summarized in Table S7.

Table S8. Summary of Kinetic Isotope Effect Experiment Results

**Substrate**	**Conversion (%)**	**Enantiomeric Excess (ee)**	**Calculated kH/kD**
**Acetophenone (-CH_3)**	15.2	>99%	1.18 ± 0.04
**Acetophenone-d_3 (-CD_3)**	13.1	97.5%	
